# A Metabolic Signature of Hereditary Transthyretin Amyloidosis: A Pilot Study

**DOI:** 10.3390/ijms232416133

**Published:** 2022-12-17

**Authors:** Marco Luigetti, Valeria Guglielmino, Angela Romano, Maria Ausilia Sciarrone, Francesca Vitali, Andrea Sabino, Jacopo Gervasoni, Aniello Primiano, Lavinia Santucci, Rossana Moroni, Guido Primiano

**Affiliations:** 1 Fondazione Policlinico Universitario Agostino Gemelli IRCCS, 00168 Rome, Italy; 2 Università Cattolica del Sacro Cuore, 00168 Rome, Italy

**Keywords:** amyloidosis, ATTRv, biomarkers, metabolites, tryptophan, palmitic acid

## Abstract

Hereditary transthyretin amyloidosis is the most common form of hereditary amyloidosis, with an autosomal dominant inheritance and a variable penetrance. ATTRv amyloidosis can present as a progressive, axonal sensory autonomic and motor neuropathy or as an infiltrative cardiomyopathy. The definition of biomarkers for the early diagnosis of ATTRv is particularly important in the current era of emerging treatments. In this sense, metabolomics could be an instrument able to provide metabolic profiles with their related metabolic pathways, and we would propose them as possible fluid biomarkers. The aim of this study is to identify altered metabolites (free fatty acids and amino acids) in subjects with a confirmed pathogenic *TTR* variant. Out of the studied total free fatty acids and amino acids, the serum values of palmitic acid are significantly lower in the ATTRv patients compared to the recruited healthy subjects. The metabolic remodeling identified in this neurogenetic disorder could be the manifestation of pathophysiological processes of the disease, such as mitochondrial dysfunction and neuroinflammation, and contribute to explaining some of its clinical manifestations.

## 1. Introduction

Hereditary transthyretin amyloidosis (ATTRv; v for variant) is the most common form of hereditary amyloidosis, with an autosomal dominant inheritance and a variable penetrance. The disease results from progressive extracellular deposition of transthyretin (TTR) amyloid fibrils, leading to progressive organ damage and death. ATTRv amyloidosis can present as a progressive, axonal sensory autonomic and motor neuropathy or as an infiltrative cardiomyopathy. The definition of biomarkers for the early diagnosis of ATTRv is particularly important in the current era of emerging treatments, which are able to significantly improve the clinical outcomes, especially when started early.

Over the last few years, the investigation of metabolic remodeling in neurodegenerative diseases, as a consequence of the underlying cellular events, has contributed to the knowledge of pathophysiological mechanisms and to the identification of disease biomarkers [1,2]. Therefore, it is evident that metabolomics can play a key role in this neurogenetic disorder in identifying specific disease-associated metabolites in order to characterize the pathways involved in metabolic adaptation and identify clinical biomarkers, a crucial aspect for the availability of disease-modifying therapies.

The aim of this study is to describe the metabolic profile in a cohort of ATTRv patients in order to identify cellular adaptation pathways and potential disease biomarkers.

## 2. Patients and Methods

This is an observational, prospective, case-control study performed at the Fondazione Policlinico Universitario Agostino Gemelli IRCCS, with the aim of describing the metabolic profile of a group of ATTRv patients and a group of healthy controls (HCs).

Blood samples were collected from 16 subjects with a confirmed pathogenic TTR variant and a diagnosis of ATTRv amyloidosis. Control samples were collected from 25 healthy volunteers without any evidence of neurologic, cardiac, renal, or autoimmune disease. The serum samples used in this study were collected in 2021–2022 and stored at –80 °C until testing. Ethics Committee of Fondazione Policlinico Universitario A. Gemelli IRCCS, Roma, Italia (protocol ID 4409), approved the study, and all participants signed an informed consent prior to inclusion. All procedures were conducted in compliance to the ethical standards laid down in the 1964 Declaration of Helsinki and its later amendments.

### 2.1. Determination of Serum Concentrations of Amino Acids and Derivatives

The serum concentration of a panel of 26 amino acids and derivatives were measured by ultraperformance liquid chromatography/MS (UPLC/MS), as described elsewhere [3,4]. The panel includes: α-aminobutyric acid, β-alanine, alanine, anserine, asparagine, aspartic acid, carnosine, cystathionine, glycine, histidine, homocitrulline, homocysteine, isoleucine, leucine, lysine, allo-isoleucine, glutamine, kynurenine, methionine, phenylalanine, proline, serine, threonine, tryptophan, tyrosine, and valine.

Briefly, 50 µL of the sample was added to 100 µL 10% (*w*/*v*) sulfosalicylic acid containing an internal standard mix (50 µM) (Cambridge Isotope Laboratories, Inc., Tewksbury, MA, USA). The mixture was centrifuged at 1000× *g* for 15 min. Then, 70 µL of borate buffer and 20 µL of AccQ Tag reagents (Waters Corporation, Milford, MA, USA) were added to 10 µL of the obtained supernatant and heated at 55 °C for 10 min. Next, samples were loaded onto a CORTECS UPLC C18 column 1.6 µm, 2.1 mm × 150 mm (Waters Corporation), for chromatographic separation (ACQUITY H-Class, Waters Corporation). Elution was accomplished at 500 µL/min flow rate with a linear gradient (9 min) from 99:1 to 1:99 water, 0.1% formic acid/acetonitrile, and 0.1% formic acid. Finally, analytes were detected on an ACQUITY QDa single-quadrupole mass spectrometer equipped with an electrospray source operating in positive mode (Waters Corporation).

The analytical process was monitored using amino acid controls (level 1 and level 2) manufactured by the MCA laboratory of the Queen Beatrix Hospital (The Netherlands). Plasma amino acid concentrations were determined by comparison with values obtained from a standard curve for each amino acid (0.5–2.5–125–250–500 μmol/L for all amino acids; only for homocystine 1–5–50–250–500–1000 μmol/L). For data analysis (calibration curves and amino acid quantitation), the instrument software TargetLynx was used.

### 2.2. Determination of Serum Fatty Acids

Ten fatty acids (tetradecanoic ac.; hexadecanoic ac.; octadecanoic ac.; cis-9-octadecenoic ac.; all cis 9,12-octadecadienoic ac.; all cis 9,12,15 octadecatrienoic ac.; all cis 8,11,14 eicosatrienoic ac.; all cis 5,8,11,14 eicosatetraenoic ac.; all cis 5,8,11,14,17 eicosapentaenoic ac.; all cis 4,7,10,13,16,19 docosahexenoic ac.) were measured in serum by gas chromatography–electron ionization–mass spectrometry (GC-EI-MS)-validated methodology (EUREKA Lab Division KIT, code GC75010; AN, ITALY).

The sample, after extraction and washing, was treated with a derivatizing for 15 min at 100 °C. The solution, after various dilutions, was directly injected into the GC-MS.

GC-MS analyses were performed using a Trace GC Ultra (Thermo Fisher Scientific, San Jose, CA, United States) equipped with Durabond HP-88 column with 100 m × 0.25 mm × 0.2 μm film thickness (Agilent, USA) and connected to a ISQ mass spectrometer (Thermo Fisher Scientific, San Jose, CA, United States). First, 1 μL of samples were injected in split mode (1:10 ratio), and the injector temperature was set at 250 °C; the carrier gas was helium, and the flow rate was maintained as constant at 1 mL/min. The initial oven temperature of 100 °C was held for 1 min, raised to 220 °C at 10 °C/min, and maintained for 4 min. After that, the oven temperature was increased to 240 °C at 10 °C/min and held for 10 min. Mass transfer line was maintained at 270 °C and the ion source at 200 °C. Analyses were performed in selected ion monitoring (SIM) mode.

The analytical process was monitored using fatty acid controls (code GC75019, level 1 and level 2) manufactured by EUREKA Lab Division (AN, ITALY).

### 2.3. Clinical and Instrumental Evaluation

All patients underwent a complete neurological and neurophysiological evaluation by an expert neurologist, and several outcome measures were assessed, including familial amyloid polyneuropathy (FAP) stage, polyneuropathy disability (PND) score, the Neuropathy Impairment Score (NIS), the Quality of Life–Diabetic Neuropathy (Norfolk QoL-DN) questionnaire, and the Compound Autonomic Dysfunction Test (CADT). Other outcome measures, such as Sudoscan, interventricular septum (IVS) thickness, and modified body mass index (mBMI), were also collected [5].

ATTRv patients were stratified according to genotype (V30M vs. non-V30M), clinical phenotype (neurologic vs. mixed), and severity of disease as evaluated by common clinical scales (FAP stage, PND score).

### 2.4. Statistical Analysis and Sample Size

The sample size of 16 ATTRv patients and 25 healthy controls was appropriate given the pilot nature of the study and its purely descriptive primary objective [6].

The sample was described in its clinical and demographic characteristics applying descriptive statistics techniques. Qualitative variables were described with absolute frequencies and percentage, and quantitative variables were summarized with median, interquartile range, mean, and standard deviation.

Kolmogorov–Smirnov test was used to assess normality of variables. Homogeneity of variance was assessed applying Levene’s test.

In order to take into account the possible effect of age and gender difference, a propensity score matching was performed considering the age as predictor and setting the match tolerance to 0.4. A final sample of 12 cases and 12 controls was obtained. Comparisons between cases and controls were performed applying the chi-square test (or the Fisher’s exact test) for categorical variables. The comparison of means for continuous, normal variables was performed with Student’s *t*-test for independent groups; not-normally distributed variables were compared with the Mann–Whitney test. As we dealt with several metabolites, Bonferroni’s correction was applied.

For those free fatty acids and amino acids whose levels were significantly different in the ATTRv patients’ group as compared to healthy controls, Pearson’s coefficient or a two-tailed Spearman’s rank-order correlation test were run to determine any linear relationship between them and demographic and clinical data in the group of ATTRv patients (disease duration, NIS, Norfolk QoL-DN questionnaire, CADT, lower and upper limbs’ Sudoscan, IVS thickness, mBMI).

Statistical analyses were performed using SPSS Statistics version 25.0.

## 3. Results

An initial total of 16 ATTRv patients and 25 healthy controls was enrolled in the study. Concerning the gender distribution, 81.3% (13/16) of ATTRv patients and 48% (12/25) of HCs were male. Mean age at evaluation was 68.75 ± 7.93 in the ATTRv patients’ group vs. 52.64 ± 10.83 in the HCs group. *t*-test highlighted a statistically significant difference in age between the two groups (*p* < 0.001). A detailed collection of demographic and clinical data of ATTRv patients is summarized in Table 1.

In order to obtain two groups with homogeneous age distribution, a propensity score matching was performed, and a final comparable sample of 12 cases and 12 controls was obtained.

Fatty acids dosage was available for all the 12 ATTRv patients and the 12 HCs (Table 2 and Supplementary Figure S1). Anserine, carnosine, cystathionine, homocitrulline, and homocystine were detectable neither in patients nor in HCs.

**Figure 1:**
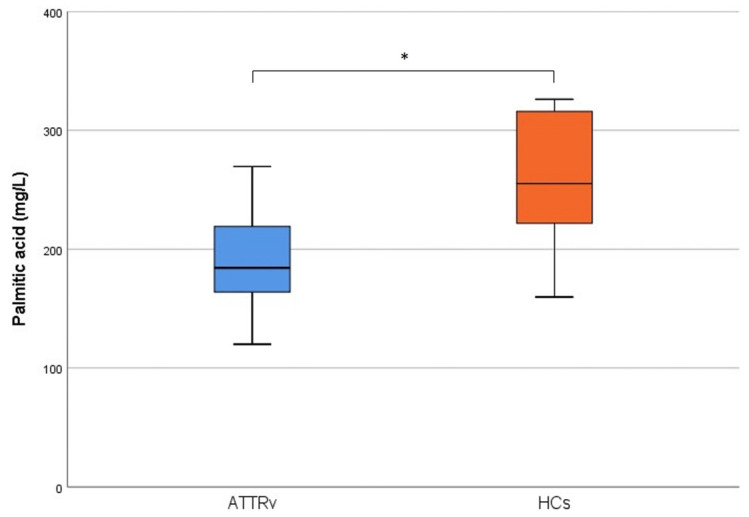
Palmitic acid levels in ATTRv patients and healthy controls, * refers to significant statistically difference (*p* = 0.002).

Palmitic acid (PA) levels were significantly lower for ATTRv patients (mean value 189.2 ± 40.6 mg/L, median 184.3, IQR (161.9–219.8) as compared to healthy volunteers (mean value 258.7 ± 56.8 mg/L, median 255.1, IQR (219.8–320.1); *p* = 0.002 (Figure 1). According to Bonferroni’s correction, a *p* < 0.005 was considered significant.

Concerning amino acids, no significant difference between ATTRv patients and HCs was found (Table 3 and Supplementary Figure S2).

As concerning ATTRv patients, comparing V30M vs. non-V30M and neurologic vs. mixed phenotypes, no significant difference was highlighted in terms of level of fatty acids and level of amino acids. The correlation analysis did not reveal any significant correlation.

## 4. Discussion

ATTRv is an autosomal dominant disease caused by the extracellular deposition of amyloid fibrils produced by misfolded transthyretin variants [7]. The natural history of the untreated disease consists of severe disability, heart failure, and death within 4–15 years from onset according to genotype. The current era of new emerging treatment changed this dramatic progression: TTR stabilizers and TTR gene-silencing therapies with small interfering RNA (siRNA) or antisense oligonucleotide (ASO) provided a therapeutic revolution, slowing the disease progression and perhaps reversing it. These innovative disease-modifying therapies are particularly effective if started early; for this reason, an early diagnosis is crucial [8].

Therefore, biomarkers are an emerging need in neurological disorders [9], especially for early diagnosis of treatable conditions such as ATTRv [10]. Considered as blood biomarkers, metabolites could be involved in detection of ATTRv disease onset and progression. Based on the analysis of ten fatty acids and twenty-six amino acids, we found that the serum values of palmitic acid in ATTRv subjects differed significantly from healthy controls.

In particular, in our cohort of patients, the blood values of palmitic acid (PA) were significantly lower compared to controls (*p* < 0.001). PA is the predominant and most common saturated fatty acid in the human body, representing 20–30% of total fatty acids in adipose triacylglycerols and in membrane phospholipids, with consequent numerous fundamental functions both at the cellular and tissue levels [11]. The concentration of this metabolite is finely regulated and controlled by a balance between dietary intake and endogenous biosynthesis from other fatty acids, carbohydrates, and amino acids [11]. The dysregulation of the homeostatic balance of PA has been associated with various physiopathological conditions, including neurodegenerative diseases. In particular, recent published articles have documented significantly decreased PA levels in subjects affected by Parkinson’s disease, hypothesizing the key role of α-synuclein in these findings [2,12]. With current evidence, it is difficult to speculate on possible common mechanisms between PD and ATTR amyloidosis underlying low serum values, referring instead to known causes of altered lipid metabolism, such as mitochondrial dysfunction [13,14,15], neuroinflammation [16], and oxidative stress [17].

ATTRv amyloidosis is due to unfolded protein accumulation, so it could be linked to an imbalance of endoplasmic reticulum (ER); the neurogenerative process of ATTRv could also be generated by ER stress, which leads to apoptosis. Wfs1 (a protein located in the ER that plays a role in the regulation the unfolded protein) mutant mice showed a downregulation in TTR expression [18], so we can speculate an interaction between TTR and Wfs1 genes.

Our study is limited due to the small number of the samples, and the obtained results will therefore be confirmed in a large cohort of patients, including pre-symptomatic carriers of ATTRv amyloidosis, in order to clarify a possible contribution of these metabolites to the pathogenesis mechanisms and their role as clinical biomarkers.

## Figures and Tables

**Table 1:** Detailed demographic and clinical data of ATTRv group.

Subject and Gender	TTR Variant	Age at Onset	Age at Evaluation	FAP Stage	PND Score	Systemic Involvement	IVS (mm)	NIS	Norfolk QoL-DN	CADT	Sudoscan LL (μS)	Sudoscan UL (μS)
**M#1**	F64L	72	75	1	2	GI	15	47,00	46	20	58	80
**M#2**	V32R	57	65	2	3b	H, Dys, K, GI	18	148,00	84	7	30	40
**M#3**	F64L	69	80	2	3a	H, Dys, GI	16	76,75	58	13	47	36
**M#4**	F64L	70	75	2	3a	H, Dys, GI	13	112,75	98	11	22	23
**M#5**	V30M	62	66	2	3a	//	10	65,00	47	17	26	56
M#6	V30M	58	66	2	3a	H, GI	13	98,00	52	18	31	45
M#7	V30M	64	69	1	2	H	15	69,50	73	17	31	71
M#8	V30M	64	75	1	1	H, GI	19	38,50	32	11	80	30
**M#9**	F64L	51	53	1	1	GI	9	28,50	18	19	76	73
**F#10**	F64L	58	60	1	1	//	10	23,00	13	15	75	79
**M#11**	F64L	63	70	2	3a	H, Dys, K, GI	22	77,75	100	15	45	67
F#12	F64L	75	75	1	1	//	12	2,00	56	13	59	71
**M#13**	V30M	54	54	1	1	H	15	12,00	2	20	76	89
**F#14**	F64L	61	69	1	2	Dys, GI	10	86,00	78	9	71	73
M#15	A109S	65	78	2	3b	Dys, GI	19	138,50	61	10	18	10
M#16	V30M	56	70	1	2	Dys, GI	17	92,00	46	11	19	24

**Legend:** TTR, transthyretin; FAP, familial amyloid polyneuropathy; PND, polyneuropathy disability score; H, heart; Dys, dysautonomia; K, kidney; GI, gastro-intestinal; IVS, interventricular septum; NIS, Neuropathy Impairment Score; Norfolk QoL-DN, Norfolk Quality of Life–Diabetic Neuropathy questionnaire; CADT, Compound Autonomic Dysfunction Test; LL, lower limbs; UL, upper limbs. In **bold** are patients selected for statistical analysis.

**Table 2:** Comparison of free fatty acids levels in ATTRv patients and in healthy controls, expressed as mean ± standard deviation (SD) and median and interquartile range (IQR). Bold values denote statistical significance at the *p* < 0.005 considering Bonferroni’s correction for multiple testing.

Free Fatty Acids (mg/L)	ATTRv Patients (*n* = 12)	Healthy Controls (*n* = 12)	*p*-Value
**Myristic acid**	2.6 ± 1.05	2.9 ± 1.6	0.496
	2.75 (1.8–3.5)	2.6 (1.7–3.8)	
**Palmitic acid**	189.2 ± 40.6	258.7 ± 56.8	**0.002**
	184.3 (161.9–219.8)	255.1 (219.8–320.1)	
**Stearic acid**	83.6 ± 24.6	78.2 ± 18.1	0.548
	80.5 (66.4–92.7)	80.8 (59.8–87.8)	
**Dihomo-γ-linoleic acid**	58.5 ± 17.9	54.6 ± 15.3	0.580
	57.5 (49.3–67.3)	56.8 (45.1–62.9)	
**Aracidonic acid**	120.3 ± 28.9	103.1 ± 26.1	0.142
	117.2 (103.1–139.8)	101.5 (81.2–117.2)	
**Oleic acid**	135.1 ± 33.5	117.1 ± 27.8	0.291
	124.7 (110.5–152.1)	123.6 (88.4–134.6)	
**Linoleic acid**	150.3 ± 50.1	122.6 ± 26.1	0.128
	144.4 (110.6–175.5)	137.2 (97.4–143.9)	
**α-Linoleic acid**	0.9 ± 0.9	103.1 ± 26.1	0.16
	0.6 (0.5–1)	101.5 (81.2–117.2)	
**Eicosapentaenoic acid (EPA)**	26.4 ± 20.2	25.2 ± 14.7	0.887
	20.5 (17.6–29.7)	22.7 (14.2–41.2)	
**Docosahexaenoic acid (DHA)**	20.5 ± 10.1	19.7 ± 9.1	0.755
	18.1 (14.7–21.1)	16.5 (13.1–30.3)	

**Table 3:** Comparison of amino acids levels in ATTRv patients and in healthy controls, expressed as mean ± standard deviation (SD) and median and interquartile range (IQR). Bold values denote statistical significance at the *p* < 0.002 considering Bonferroni’s correction for multiple testing.

Amino Acids (micromol/L)	ATTRv Patients (*n* = 12)	Healthy Controls (*n* = 12)	*p*-Value
**α-aminobutyric acid**	17.7 ± 7.9	22.1 ± 8.1	0.215
	16.1 (11.8–23.1)	19.4 (16.6–27.2)	
**Asparagine**	45.6 ± 9.6	51.5 ± 10.5	0.184
	46 (38.4–53.3)	51.9 (40.3–60.7)	
**Aspartic acid**	28.5 ± 10.8	22.8 ± 7.4	0.179
	31.8 (18.–35.6)	19.3 (16.6–31.1)	
**Isoleucine**	7.9 ± 18.8	66.2 ± 12.8	0.285
	75.5 (59.6–88.3)	65.0 (56.0–78.6)	
**Leucine**	143.5 ± 32.9	132.2 ± 24.5	0.379
	150.1 (115.0–172.4)	127.6 (114.8–151.9)	
**Lysine**	201.5 ± 46.5	212.4 ± 31.7	0.537
	188.7 (162.1–251.4)	200.1 (188.4–238.8)	
**Methionine**	23.6 ± 7.9	24.5 ± 4.4	0.726
	22.3 (17.9–31.1)	24.8 (21.1–27.7)	
**Phenylalanine**	74.8 ± 18.8	71.1 ± 11.1	0.595
	79.6 (61.3–90.2)	69.7 (62.7–80.2)	
**Proline**	281.33 ±110.8	197.5 ± 47.7	0.031
	284.9 (190.5–358.4)	182.7 (160.1–238.2)	
**Serine**	140.9 ± 25.1	129.1 ± 28.5	0.314
	138. (131.2–163.1)	128.0 (100.2–153.3)	
**Tryptophan**	55.1 ± 12.1	61.0 ± 8.3	0.207
	54.3 (43.1–65.8)	59.3 (55.2–64.8)	
**Tyrosine**	64.9 ± 21.4	67.9 ± 20.6	0.744
	59.8 (47.1–89.9)	66.3 (52.5–84.1)	
**Valine**	240.2 ± 48.2	237.4 ± 41.8	0.884
	234.1 (199.2–288.7)	231.4 (205.4–268.1)	
**Allo-Isoleucine**	1.6 ± 0.6	1.6 ± 0.5	0.766
	1.5 (1.1–1.9)	1.5 (1.2–2.2)	
**Alanine**	529.2 ± 386.3	426.7 ± 117.6	0.923
	414.7 (337.1–535.0)	427.6 (377.9–493.7)	
**β-alanine**	10.8 ± 4.6	11.3 ± 9.7	0.314
	9.6 (7.7–12.3)	7.1 (5.3–15.2)	
**Glycine**	303.8 ± 130.2	264.1 ± 50.8	0.923
	249.1 (231.9–337.8)	260.5 (230.0–293.8)	
**Histidine**	84.1 ± 47.4	79.9 ± 14.7	0.771
	64.5 (60.4–99.2)	83.8 (62.–90.8)	
**Threonine**	135.3 ± 45.0	134.3 ± 24.7	0.628
	121.7 (109.0–153.9)	126.8 (112.6–157.5)	
**Glutamine**	684.1 ± 300.6	718.1 ± 292.1	0.582
	585.3 (439.3–991.9)	634.9 (530.9–782.9)	
**Kyneurine**	3.44 ± 1.3	3.1 ± 0.4	0.771
	2.9 (2.7–4.1)	3.1 (2.7–3.4)	

## Data Availability

The data that support the findings of this study are available from the corresponding author, upon reasonable request.

## Supplementary Materials

The following supporting information can be downloaded at: https://www.mdpi.com/article/10.3390/ijms232416133/s1, Supplementary Figure S1; Supplementary Figure S2.
