# Supplementary figures and images for: A Metabolic Signature of Hereditary Transthyretin Amyloidosis: A Pilot Study
Source: Int J Mol Sci. 2022 Dec 17;23(24):16133. doi: 10.3390/ijms232416133 (PMC9783933; doi:10.3390/ijms232416133)

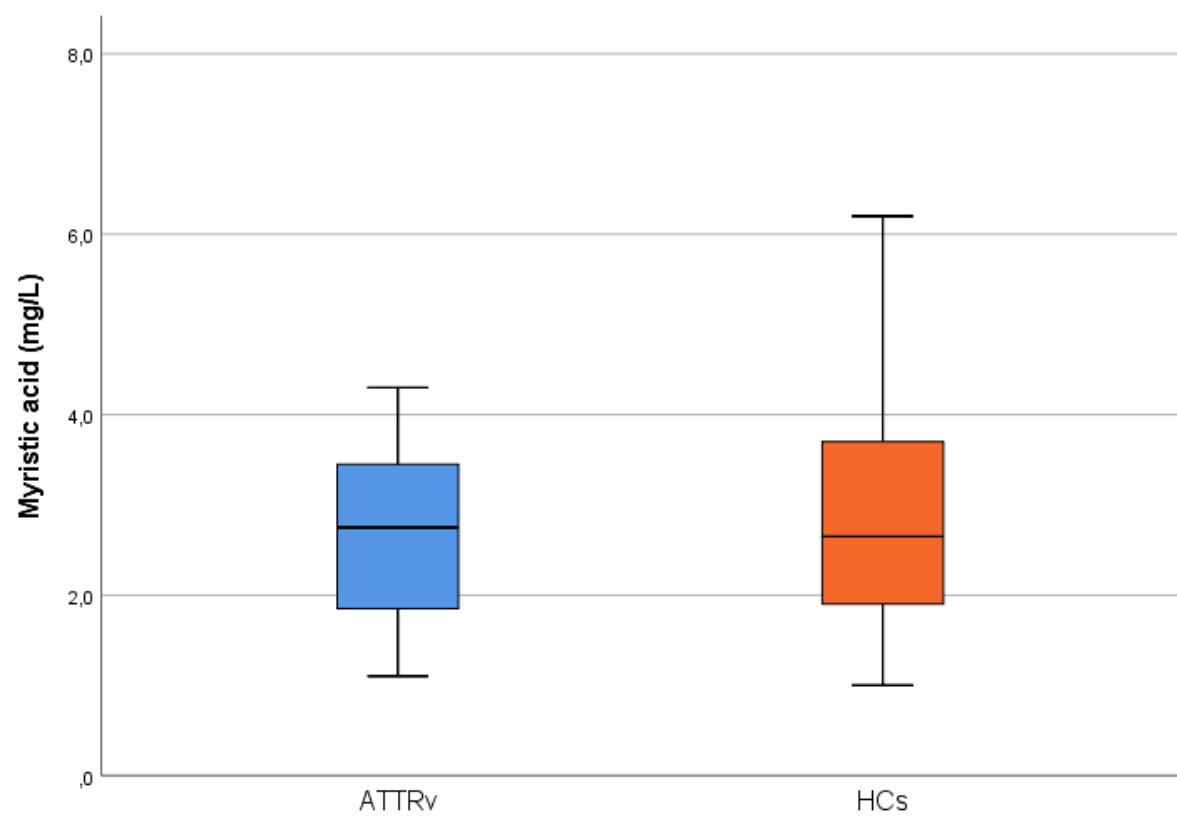

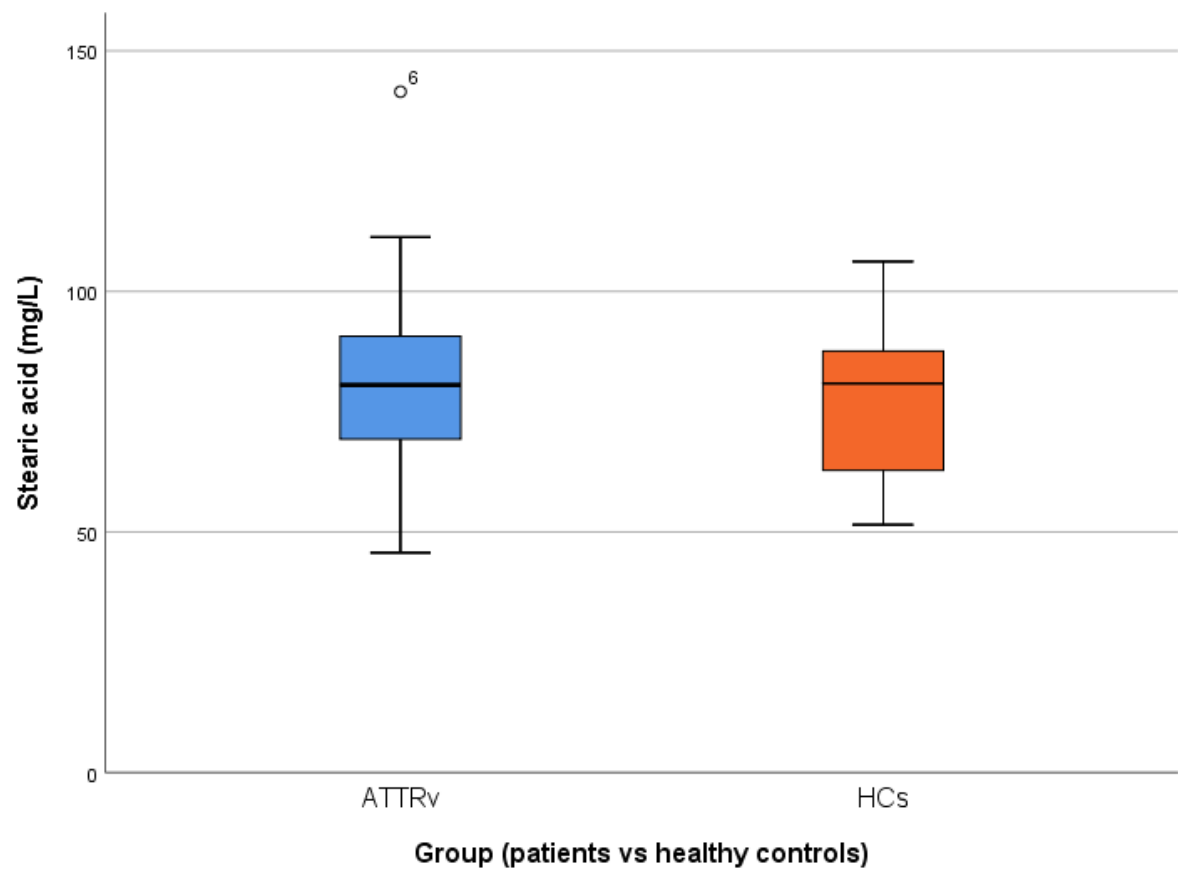

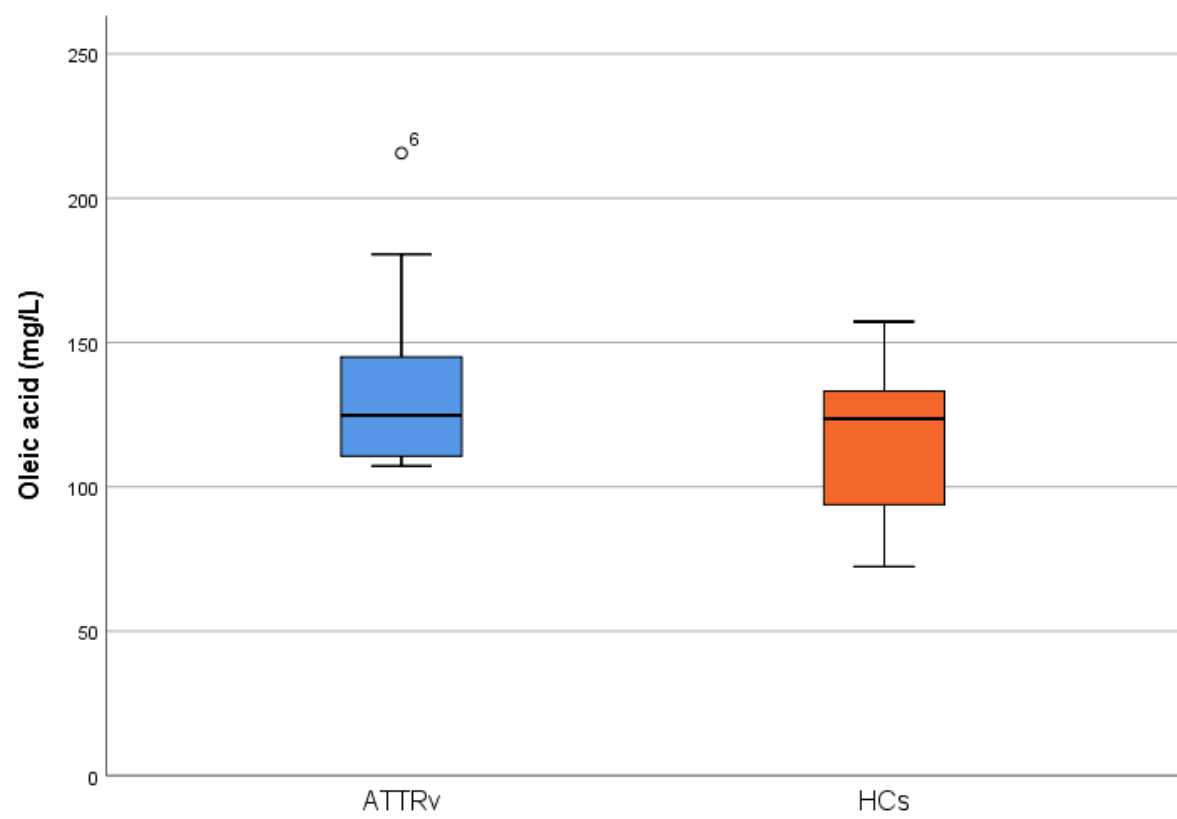

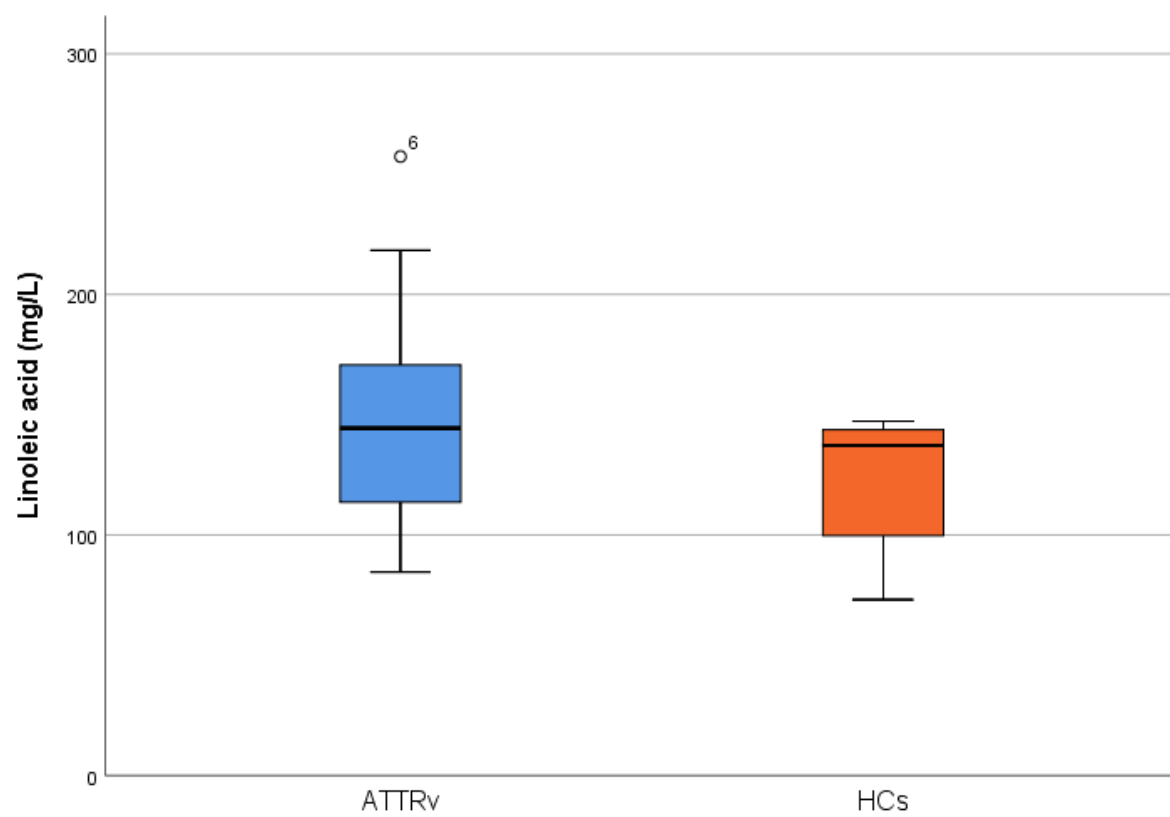

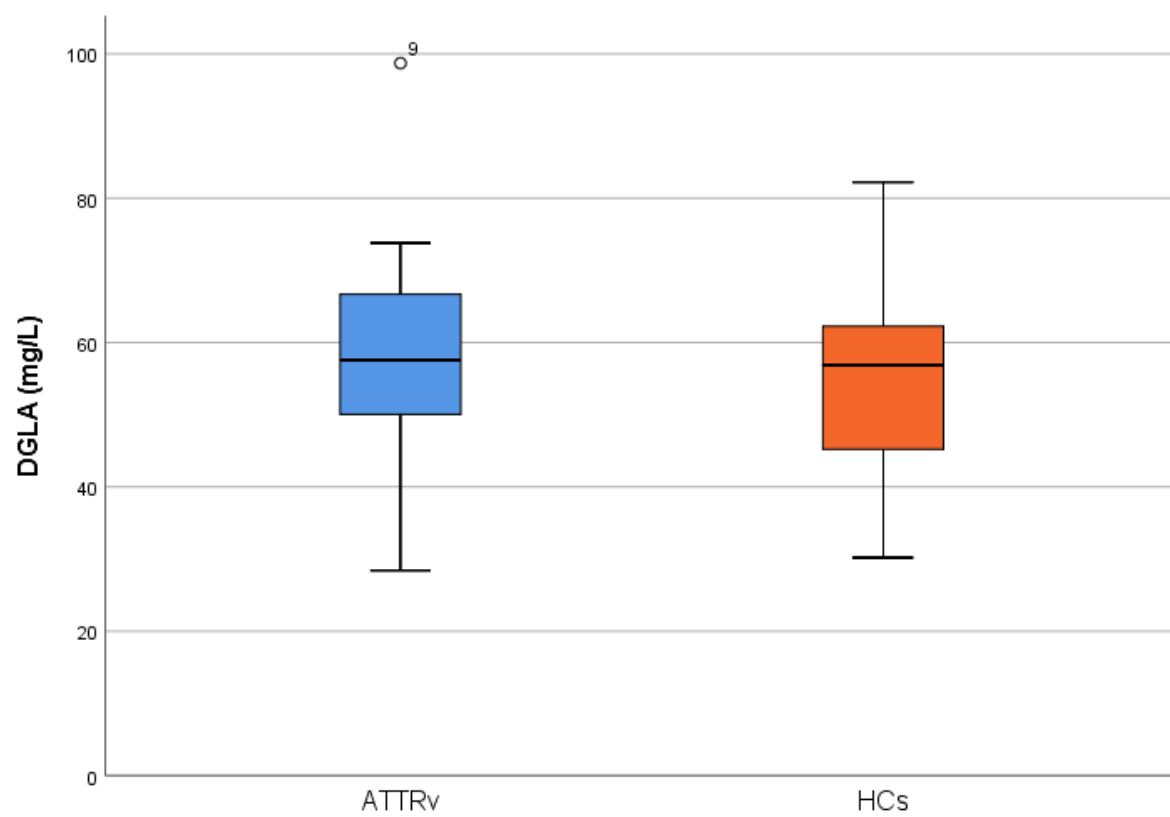

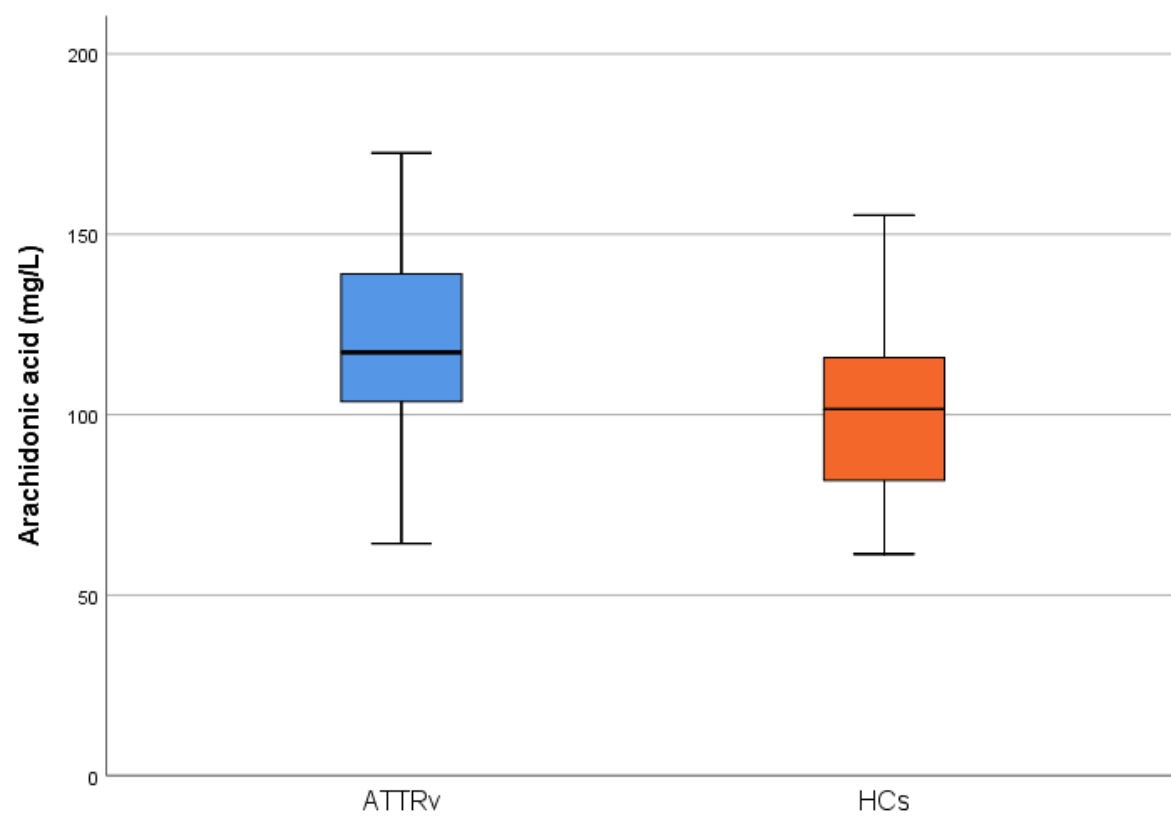

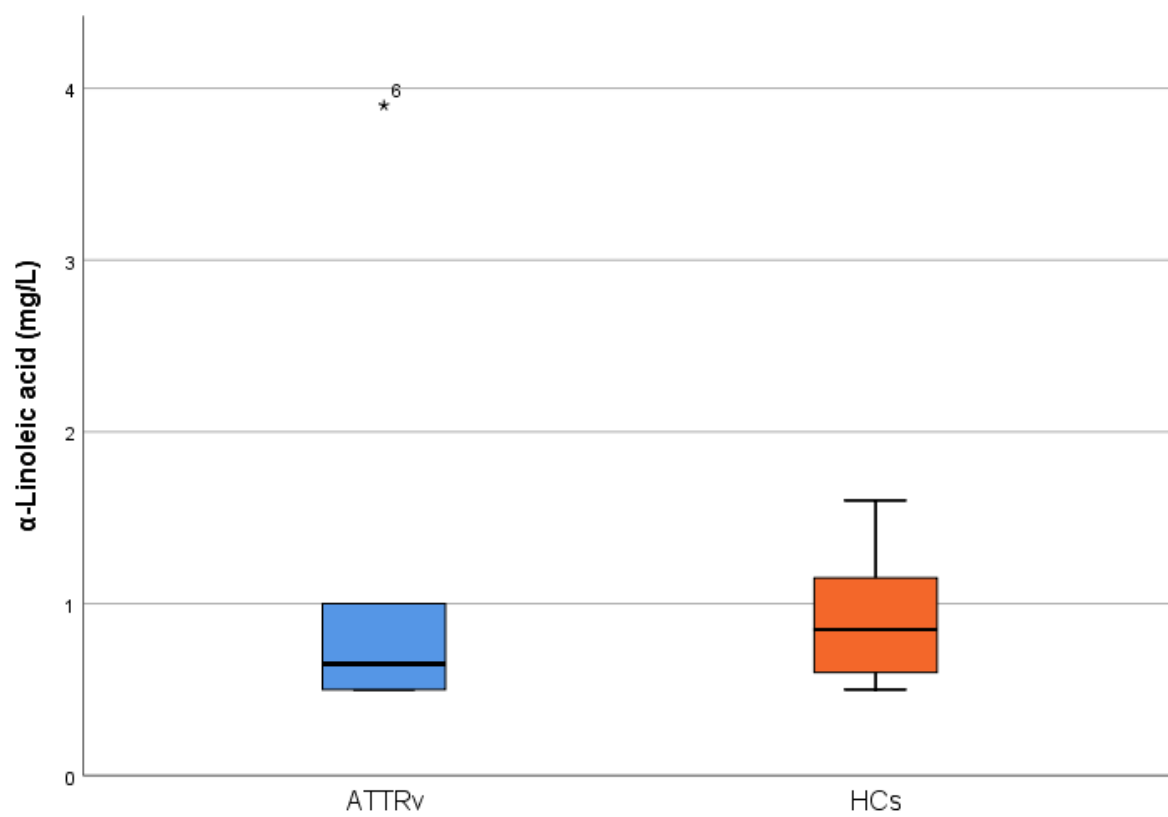

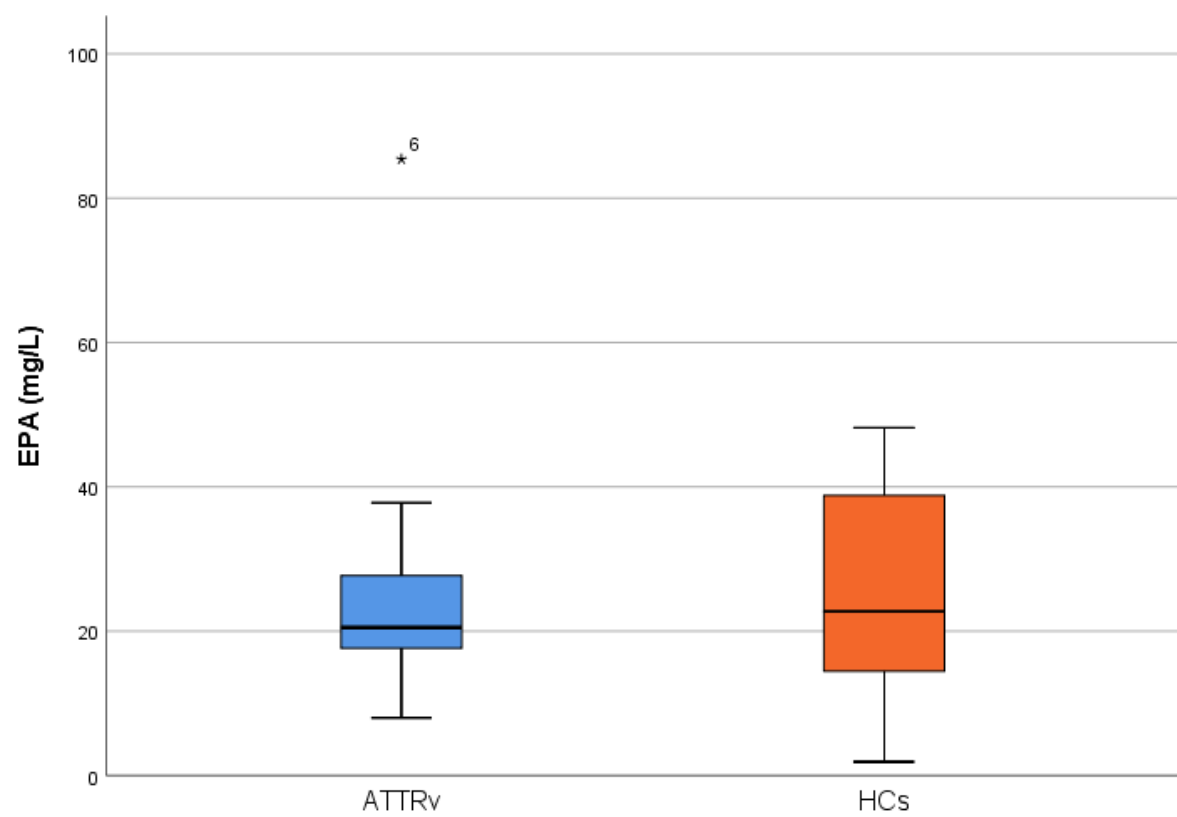

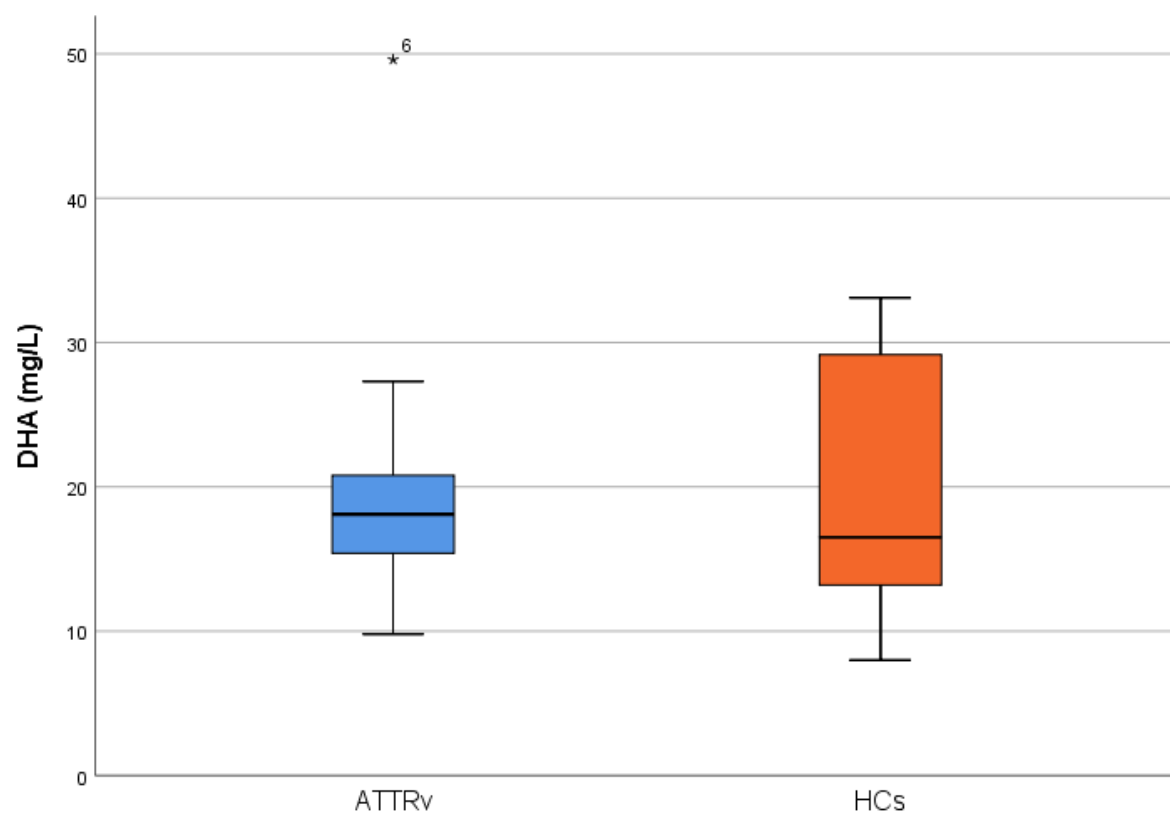

Supplement: Supplementary file 1 [file ijms-23-16133-s001.zip › Supplementary figure S1.pdf]

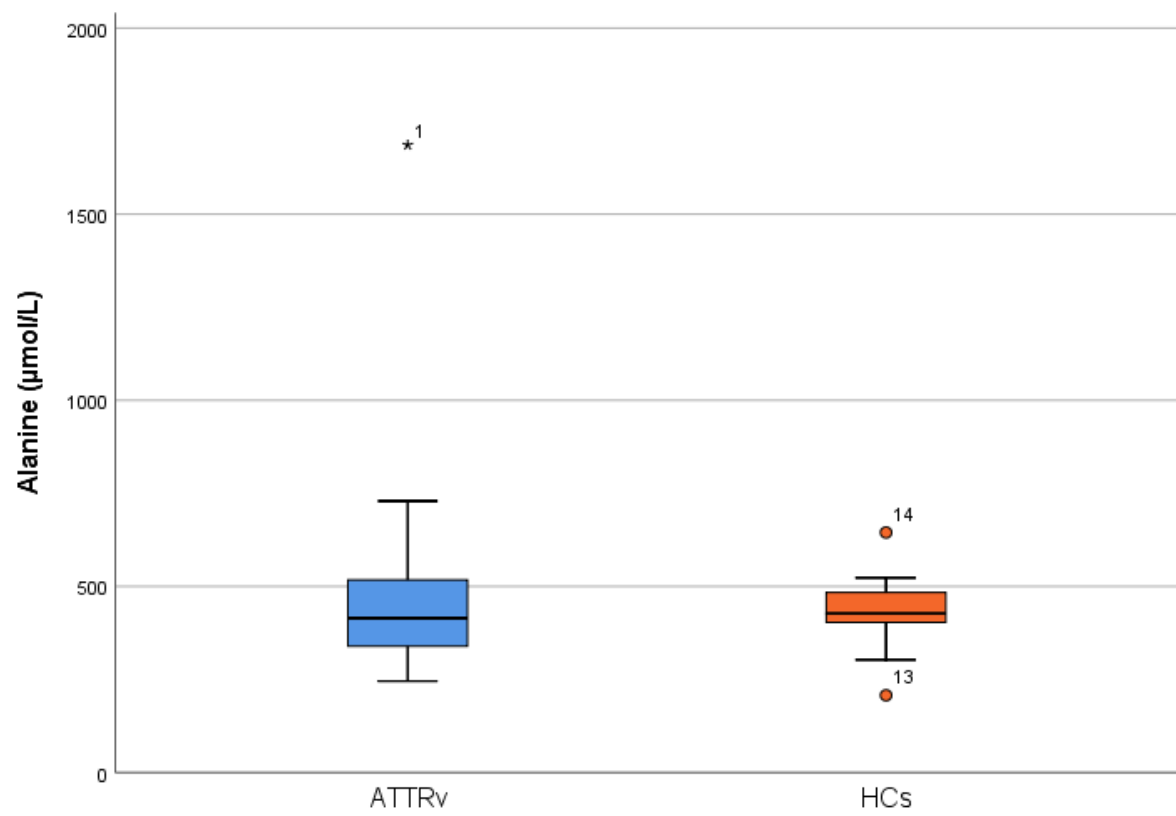

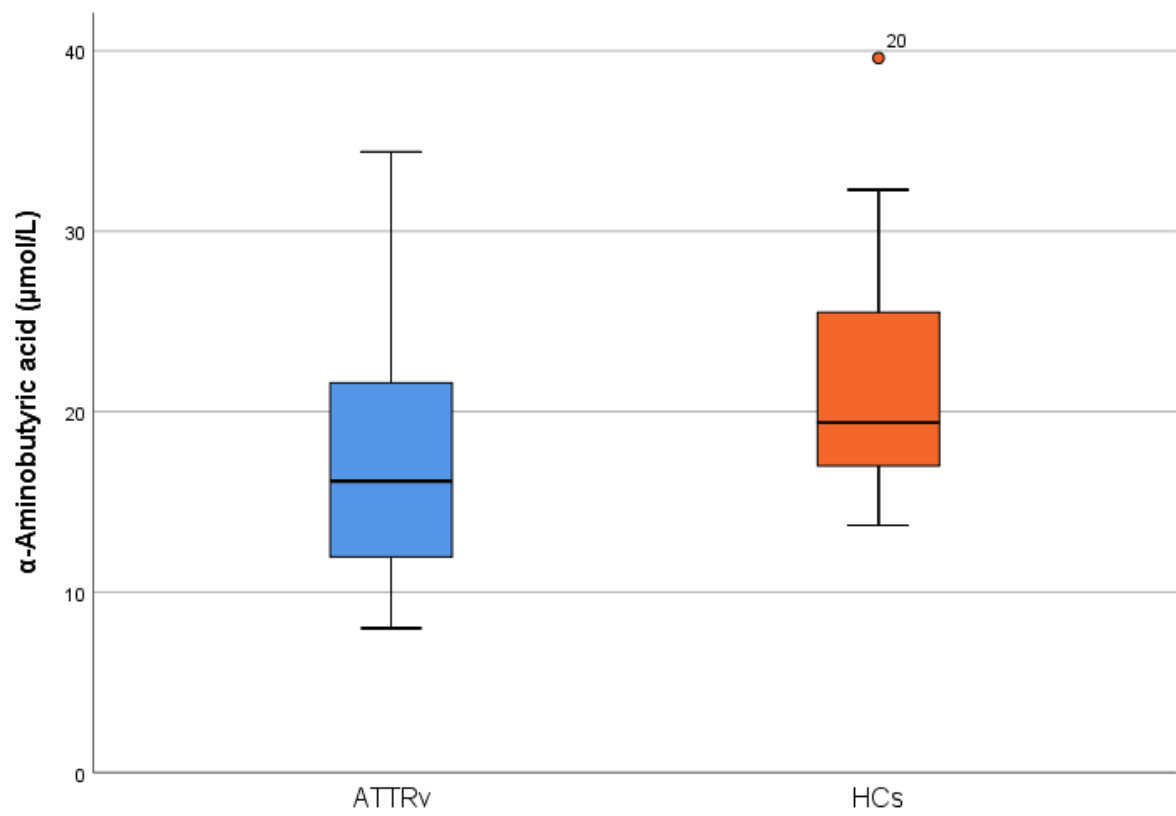

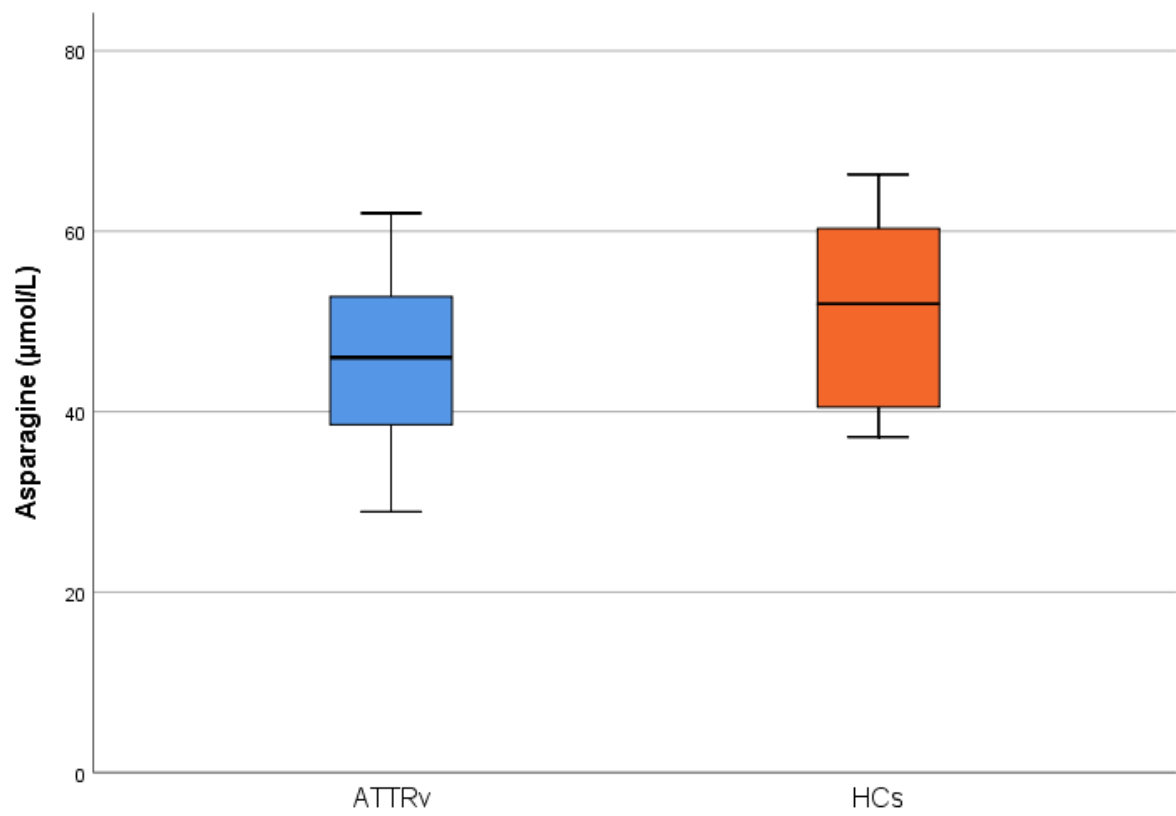

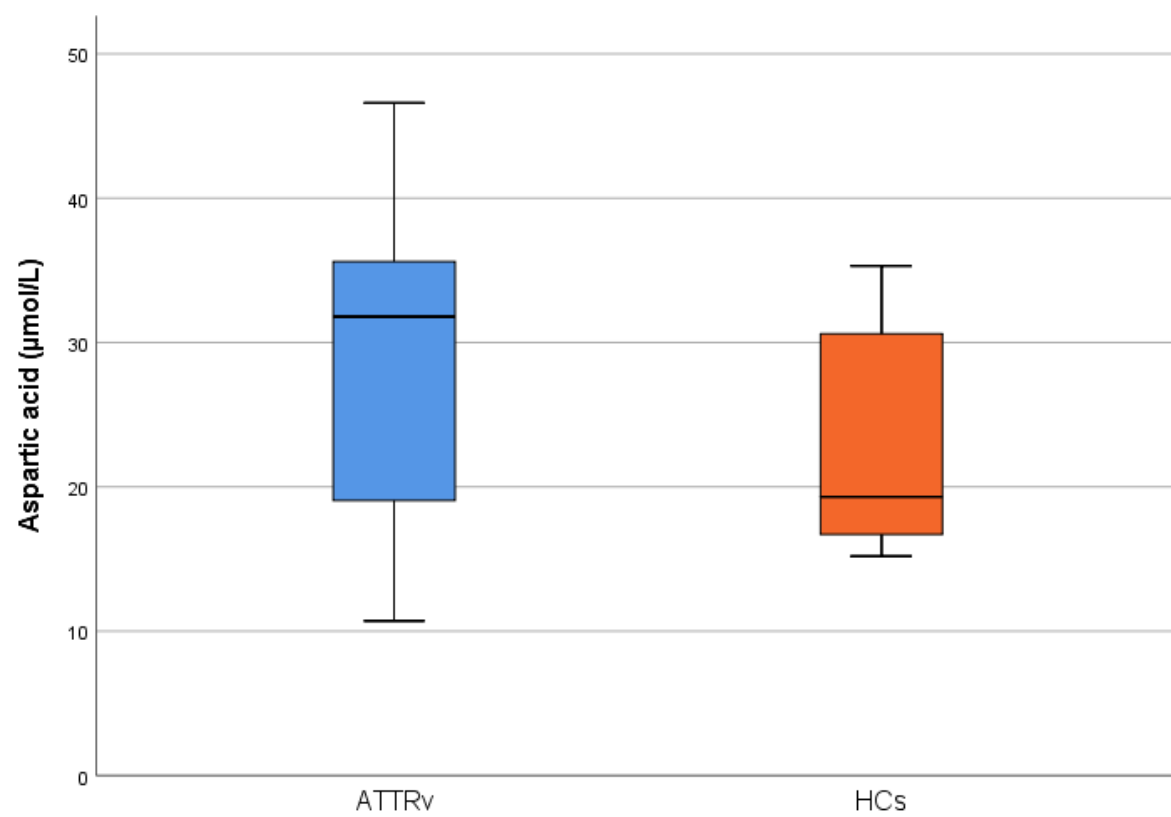

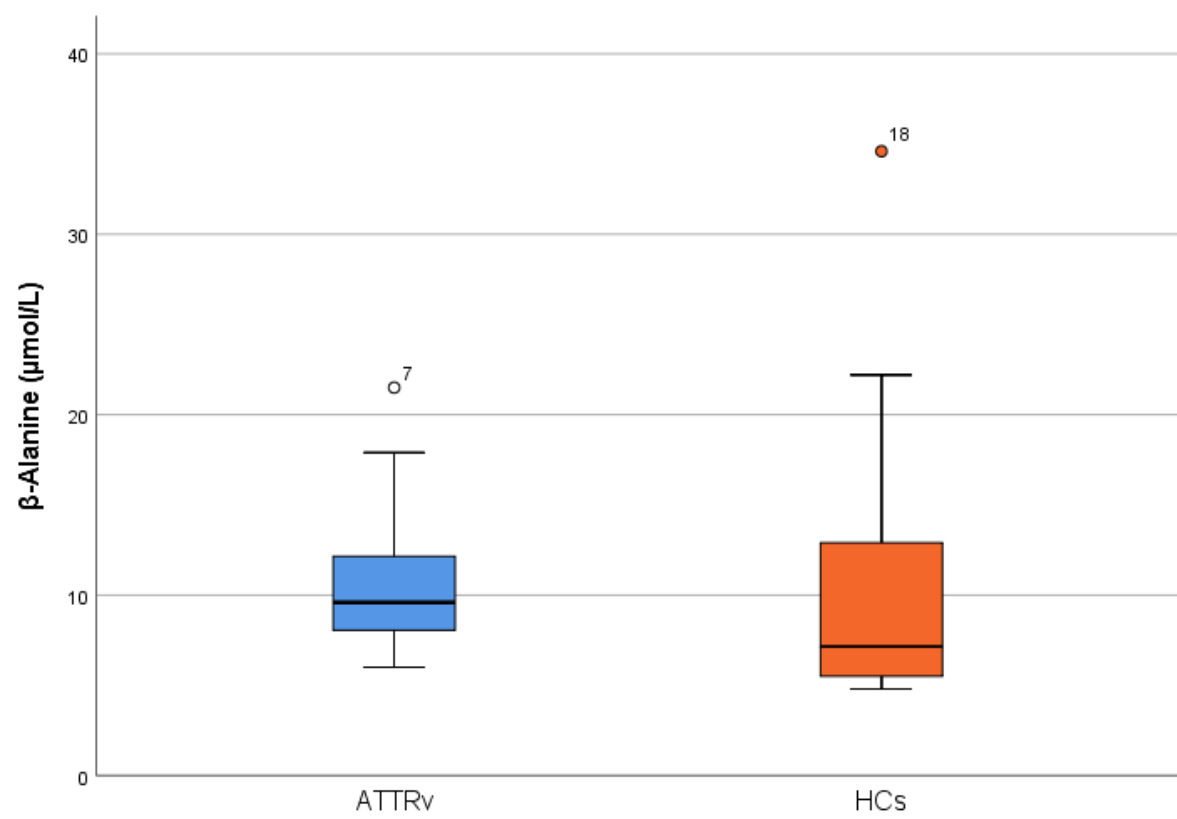

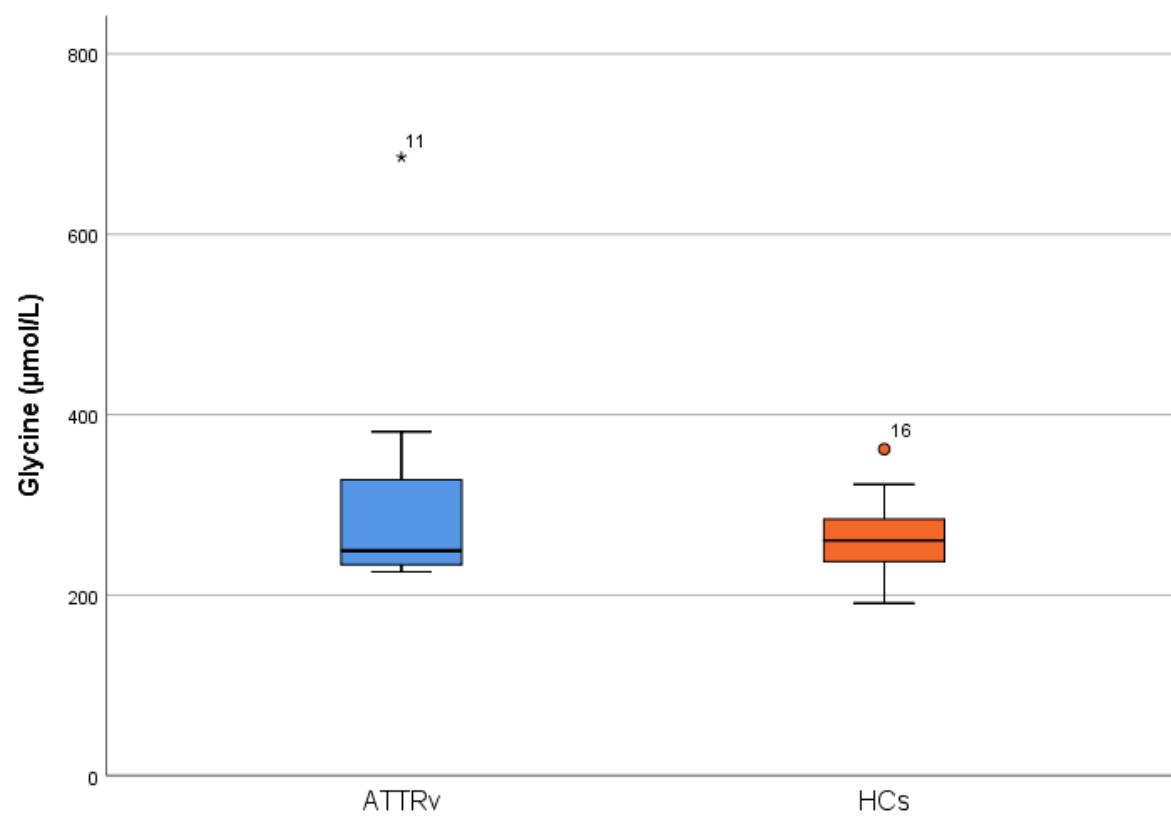

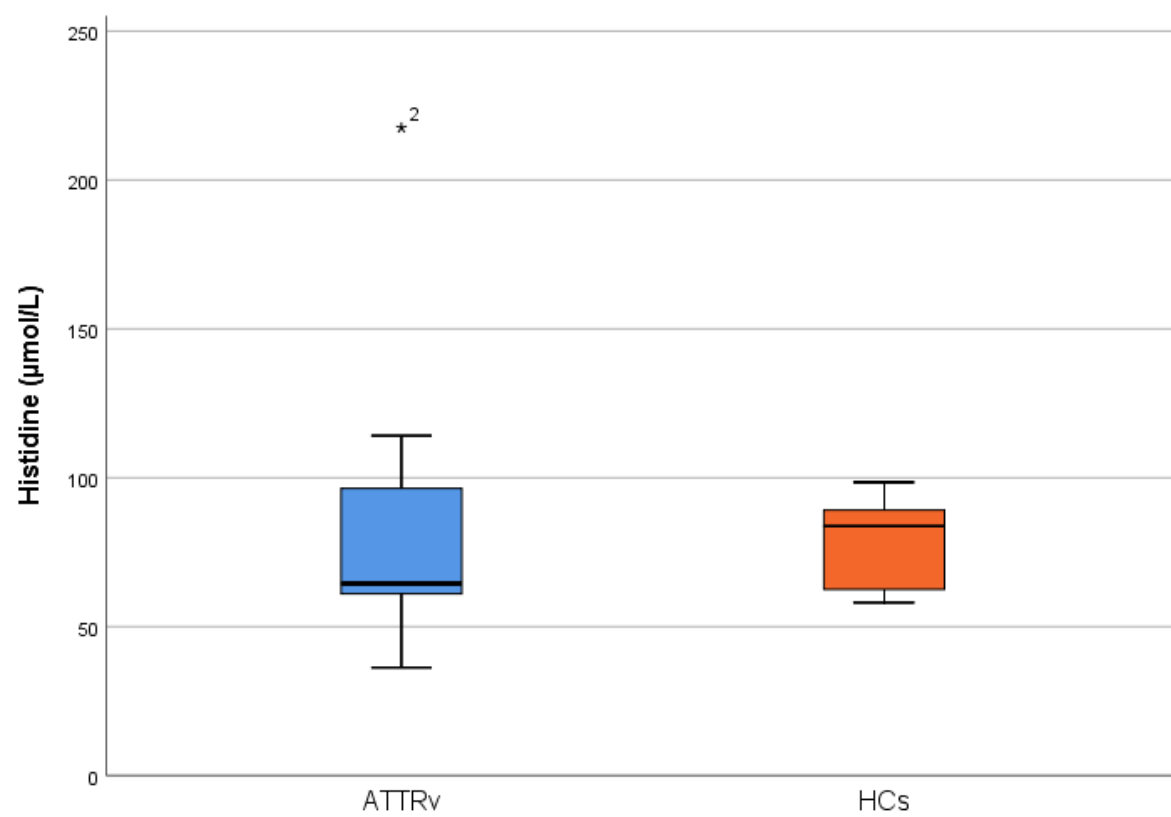

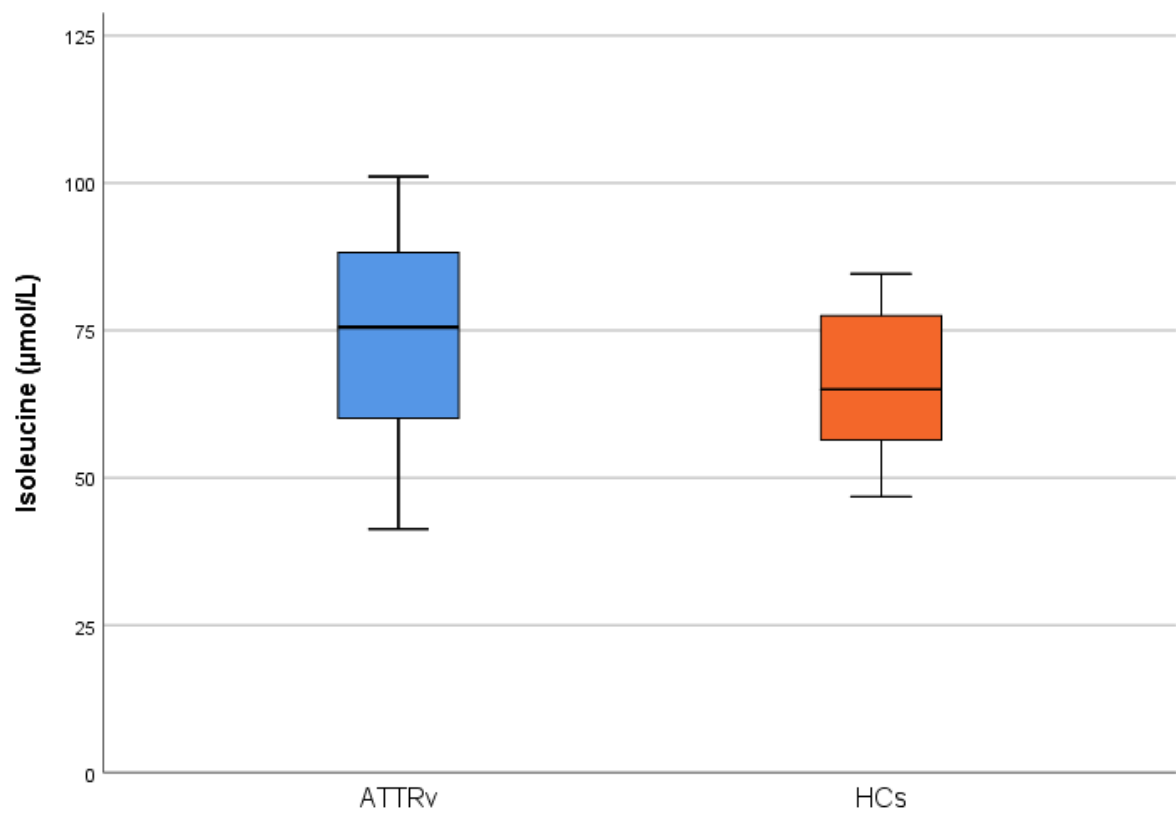

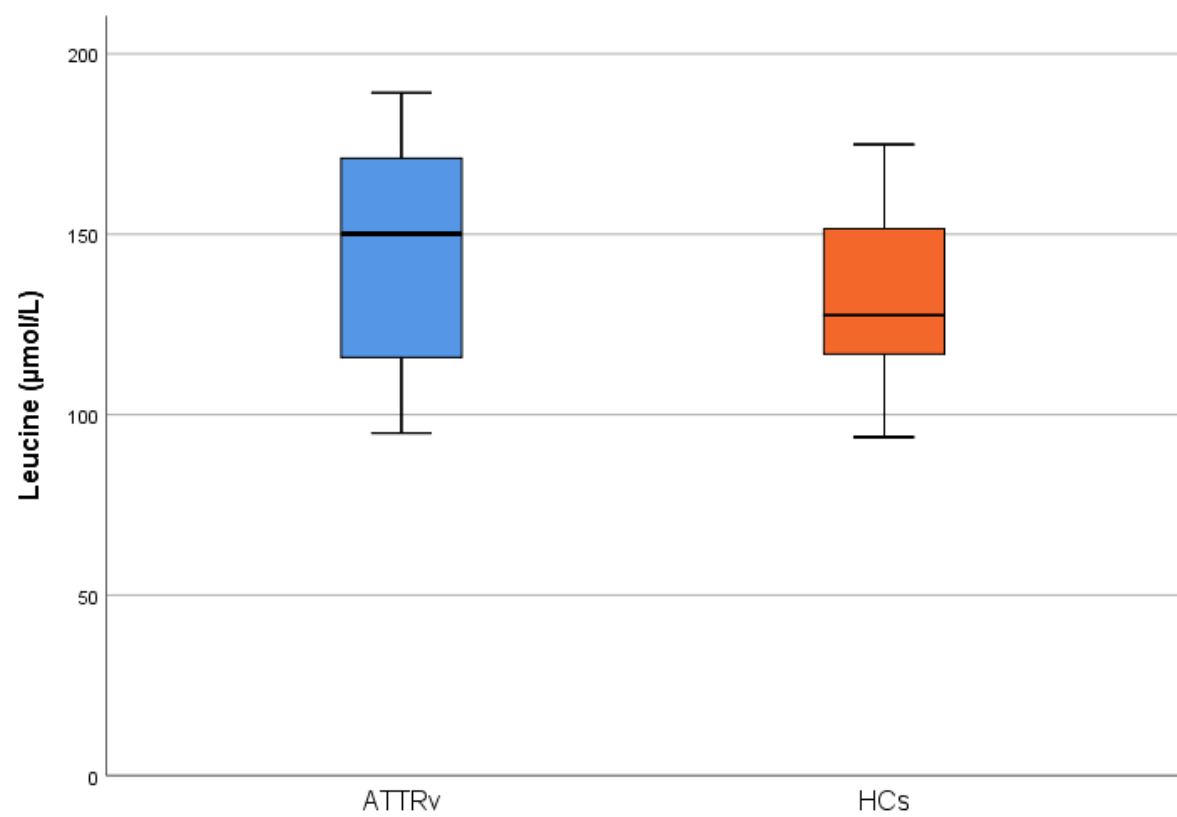

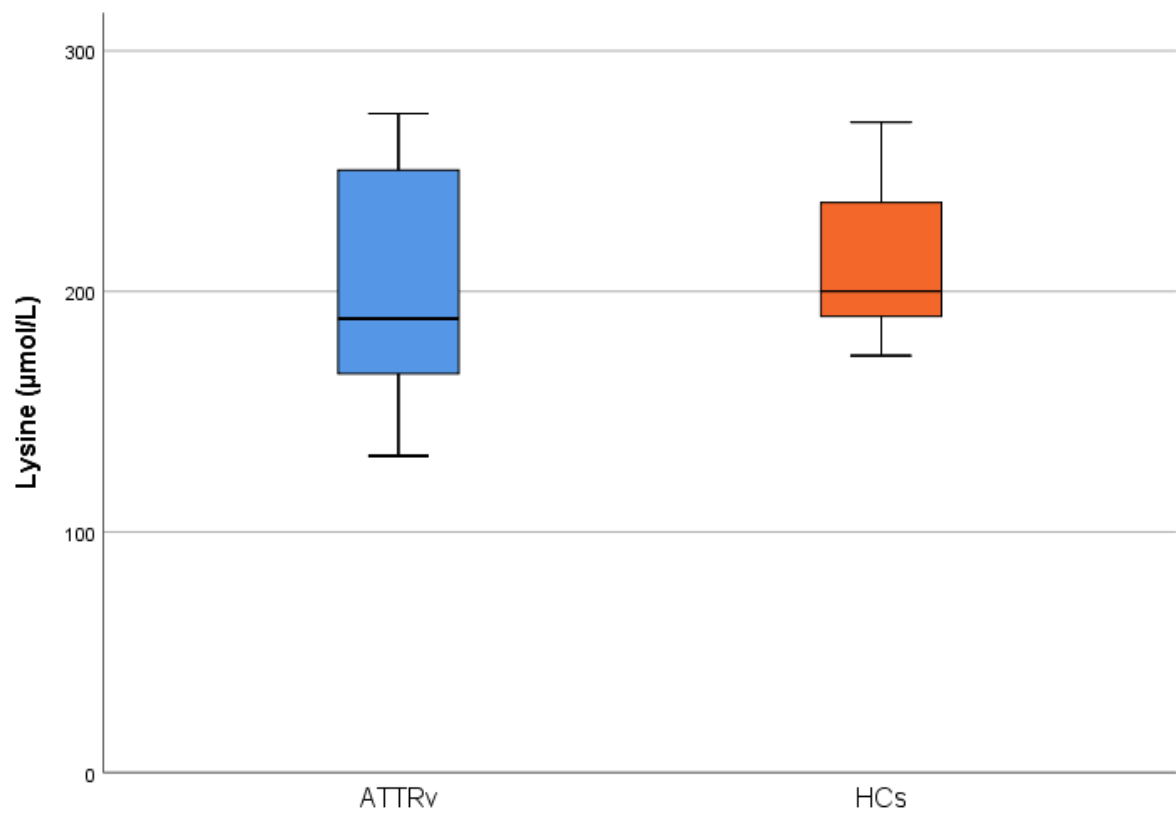

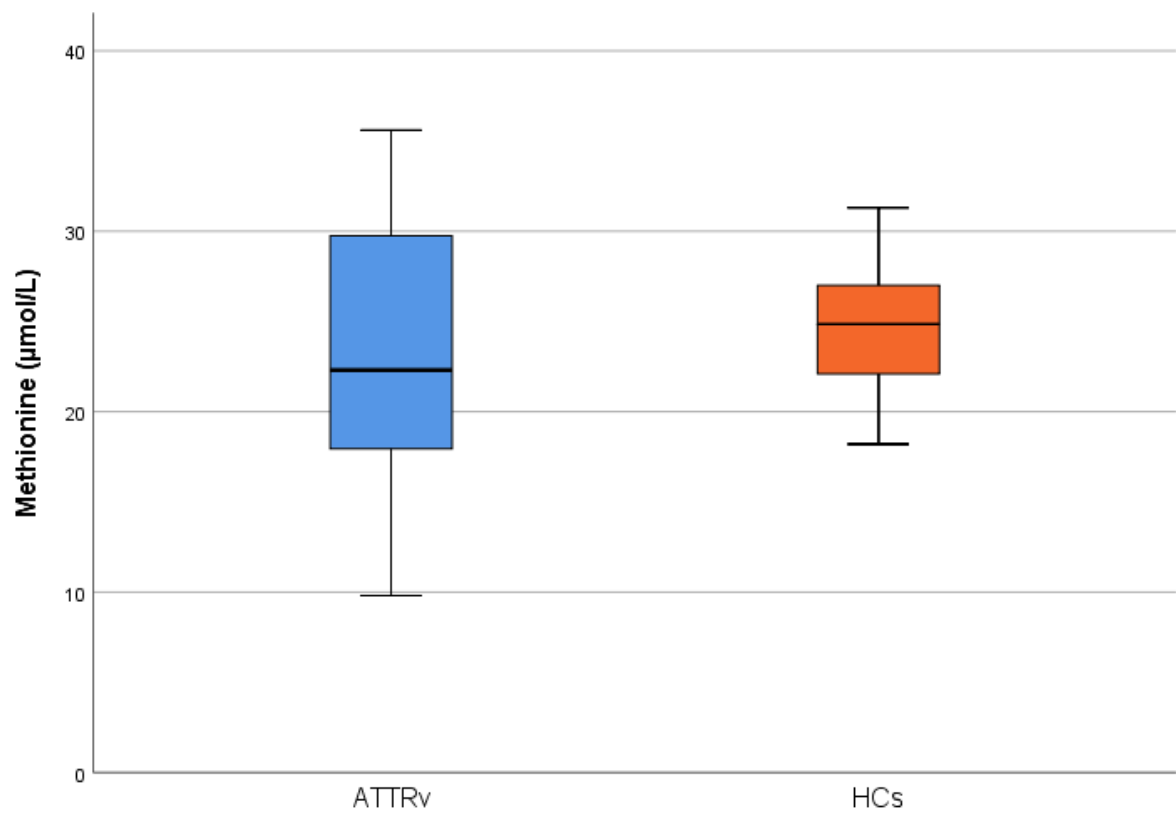

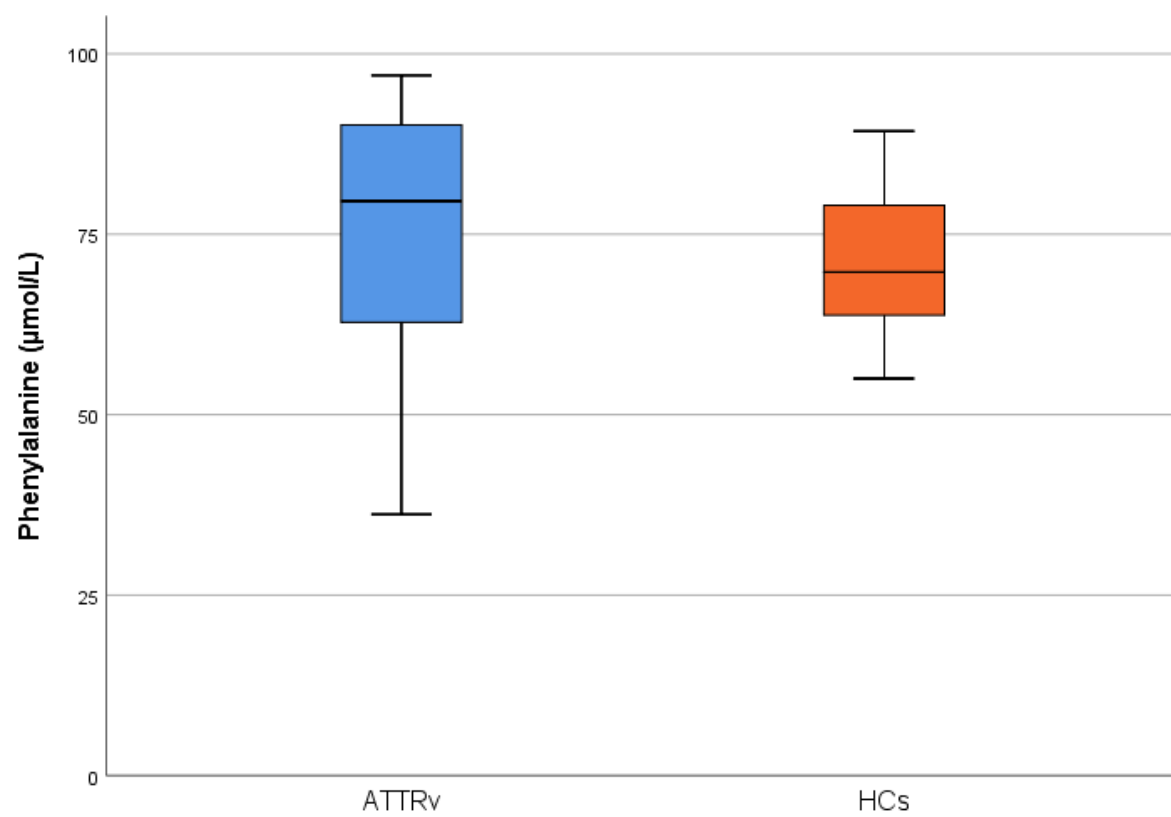

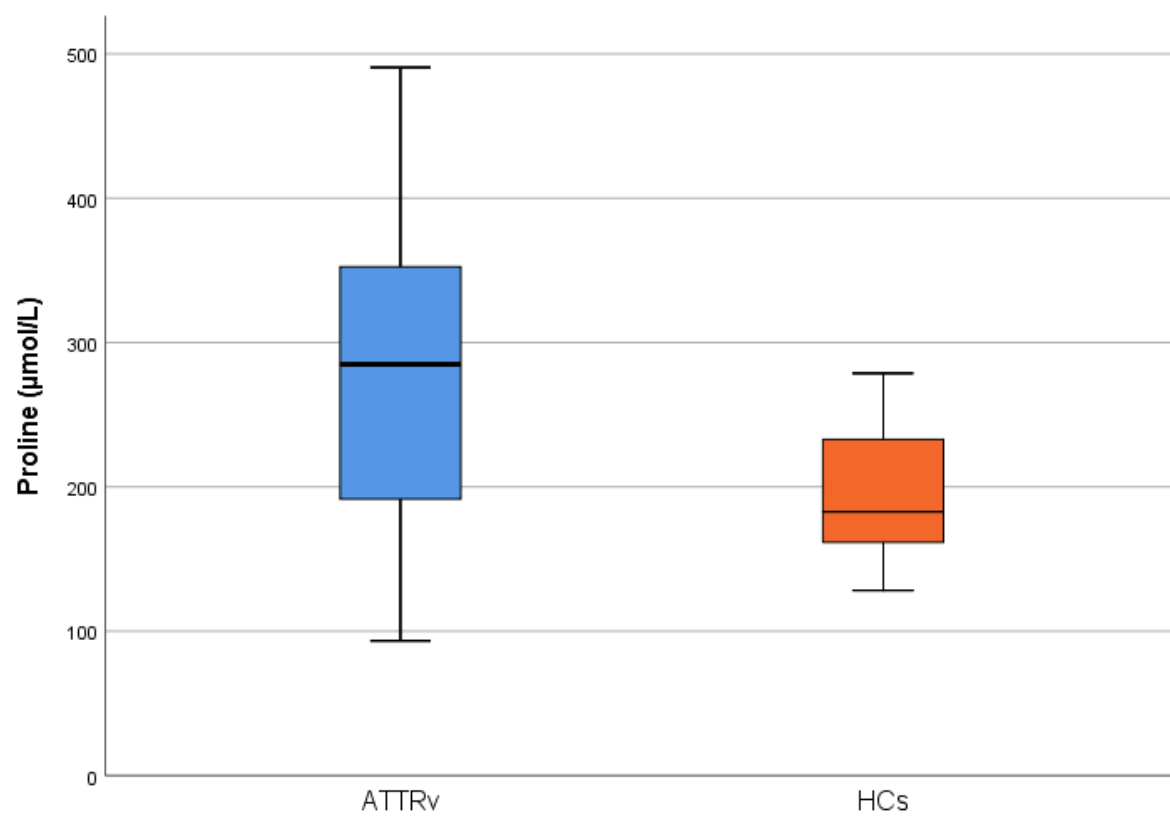

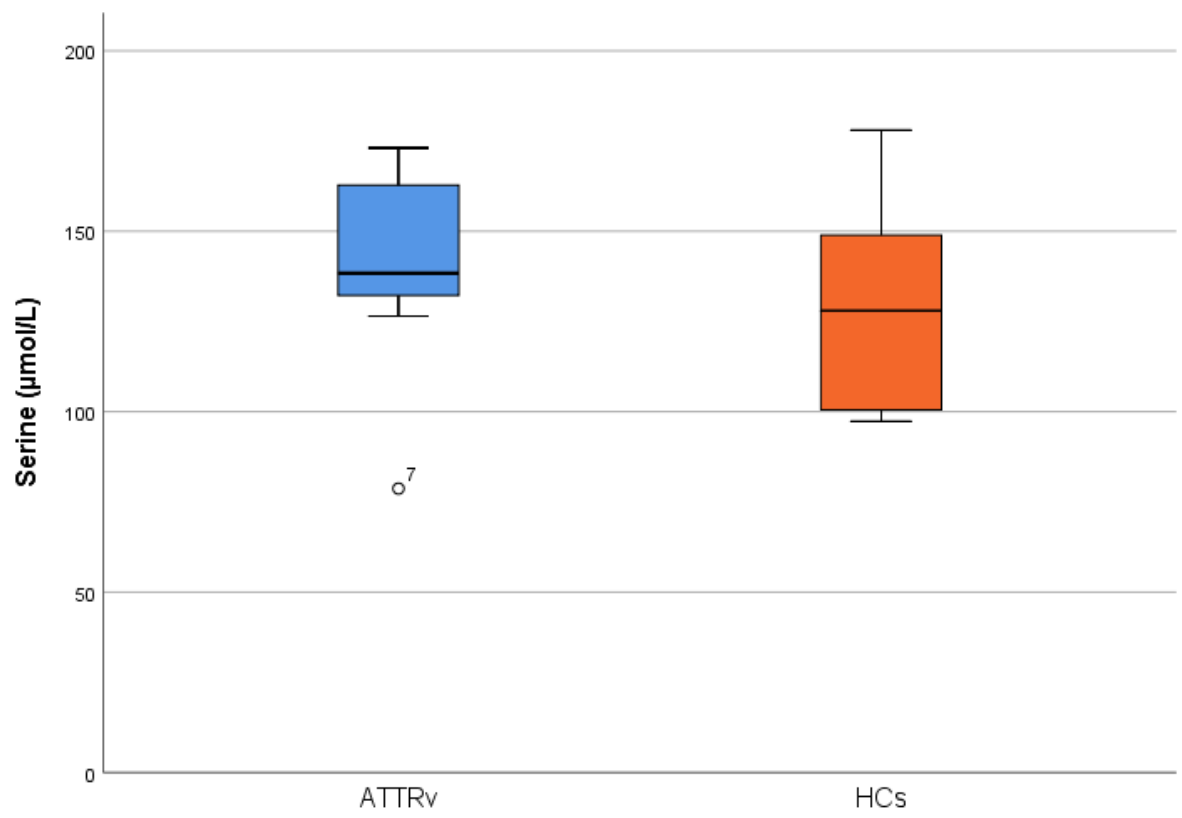

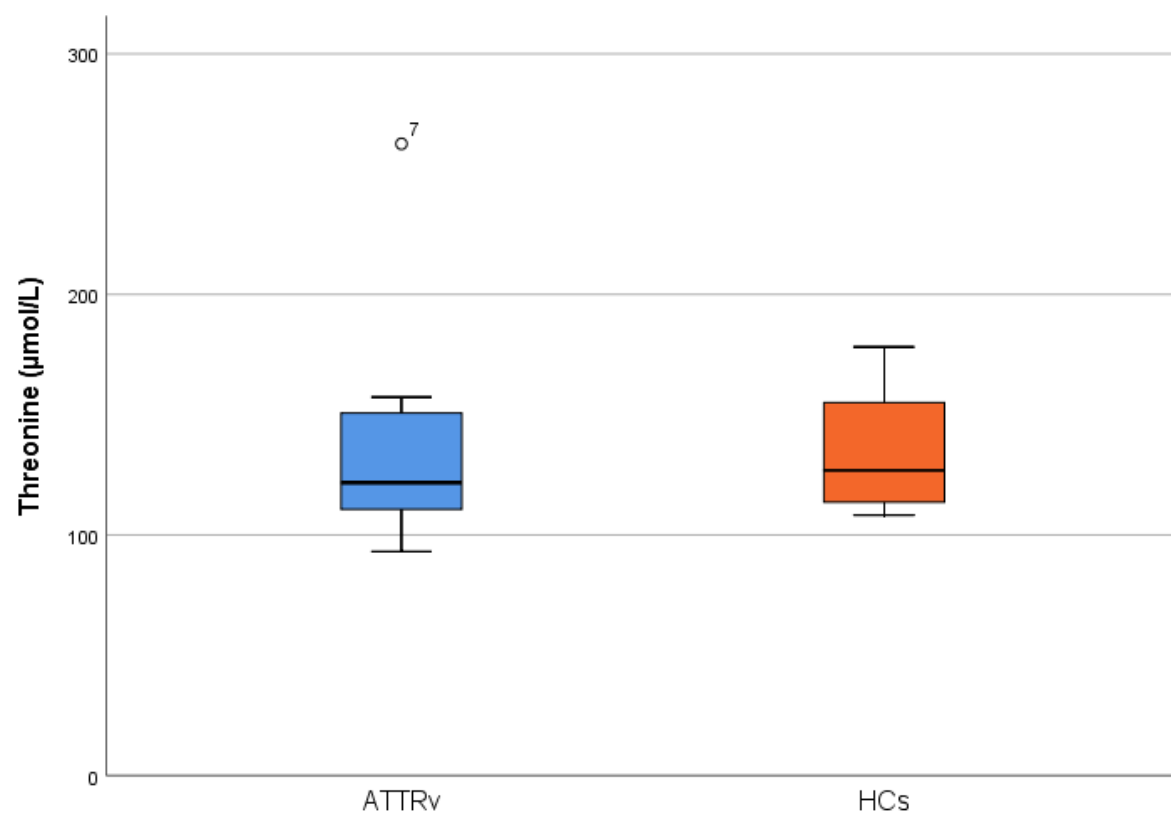

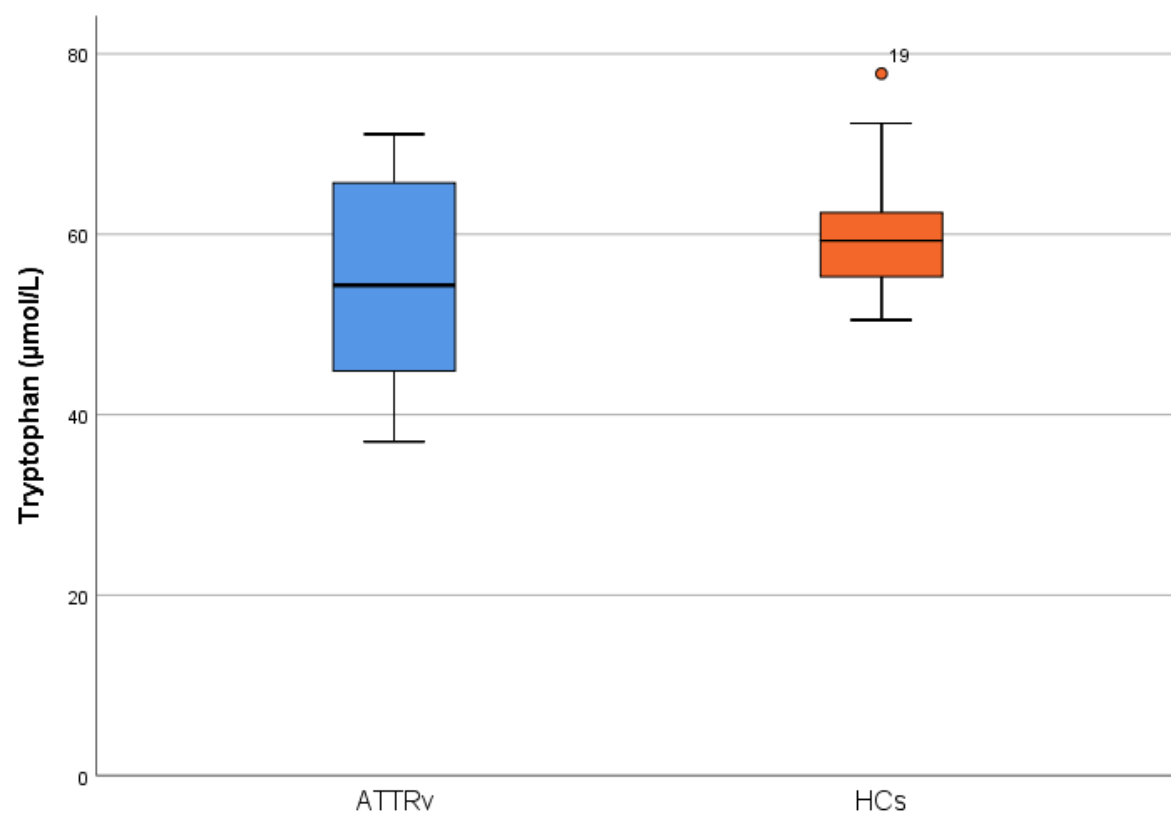

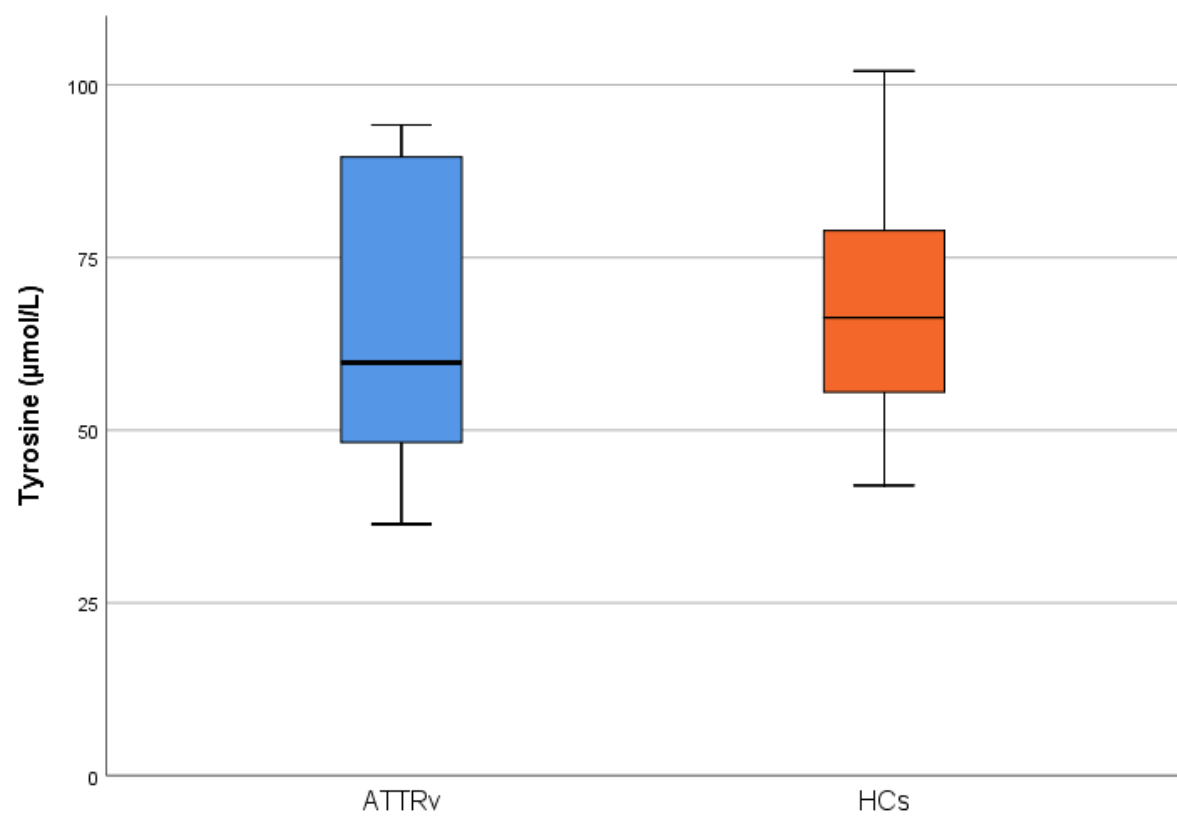

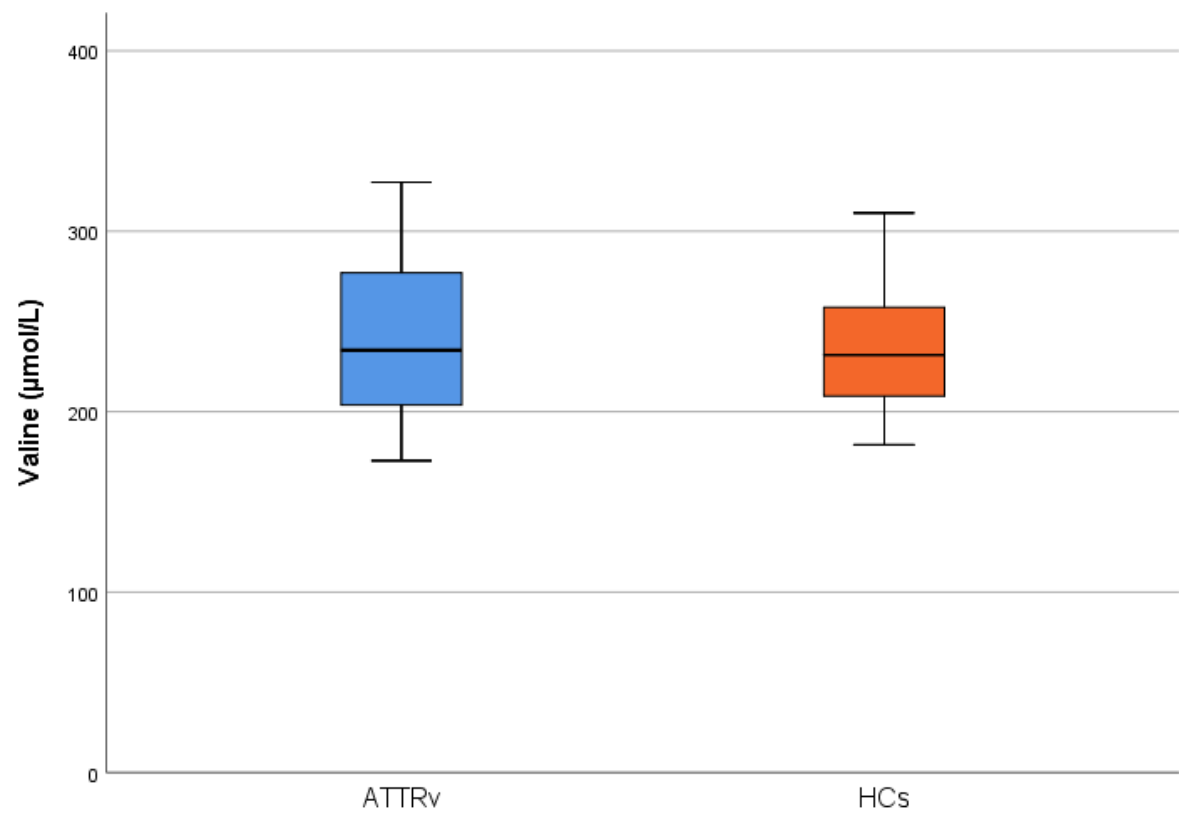

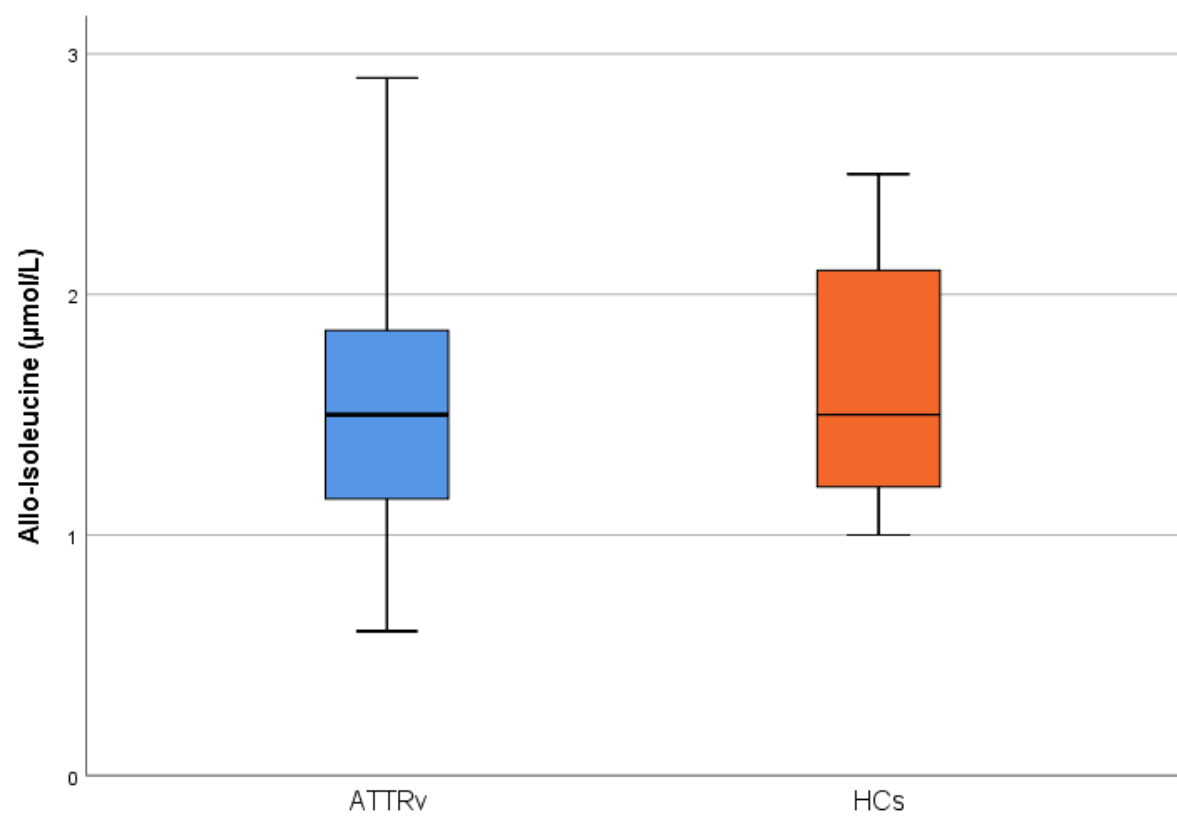

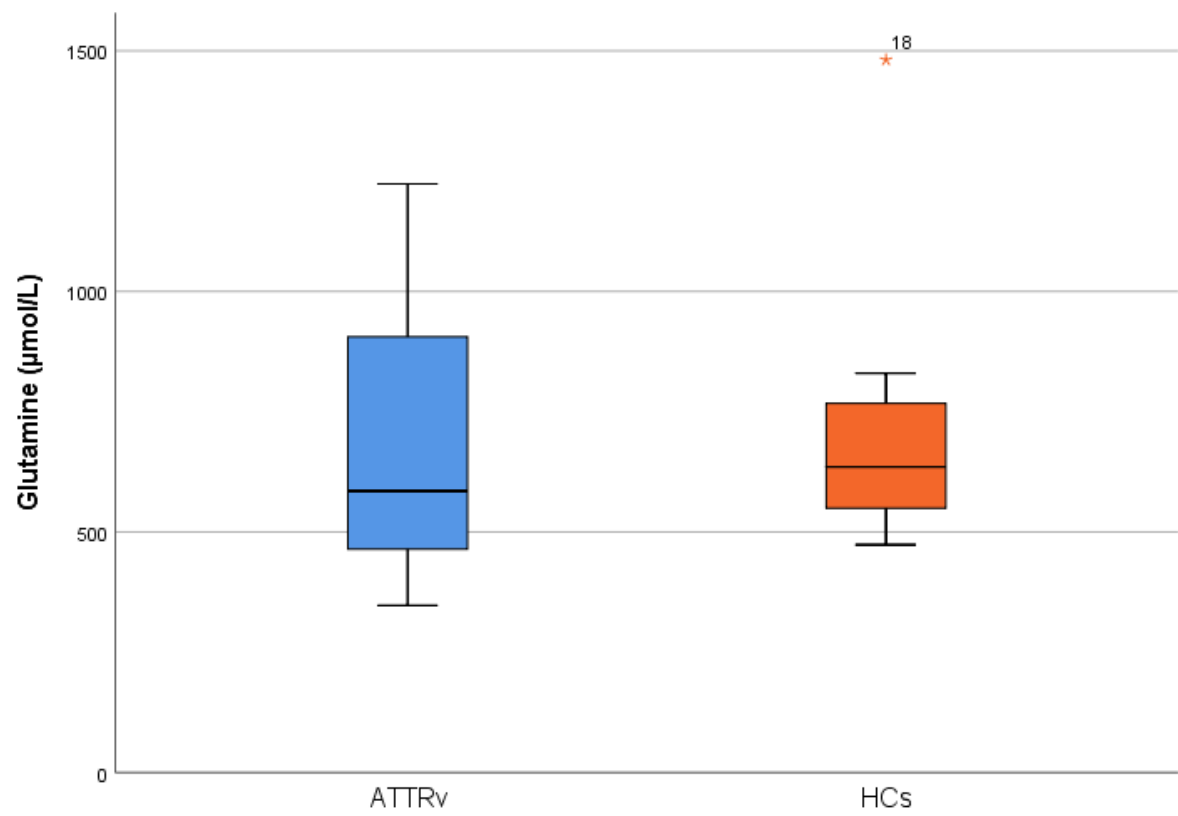

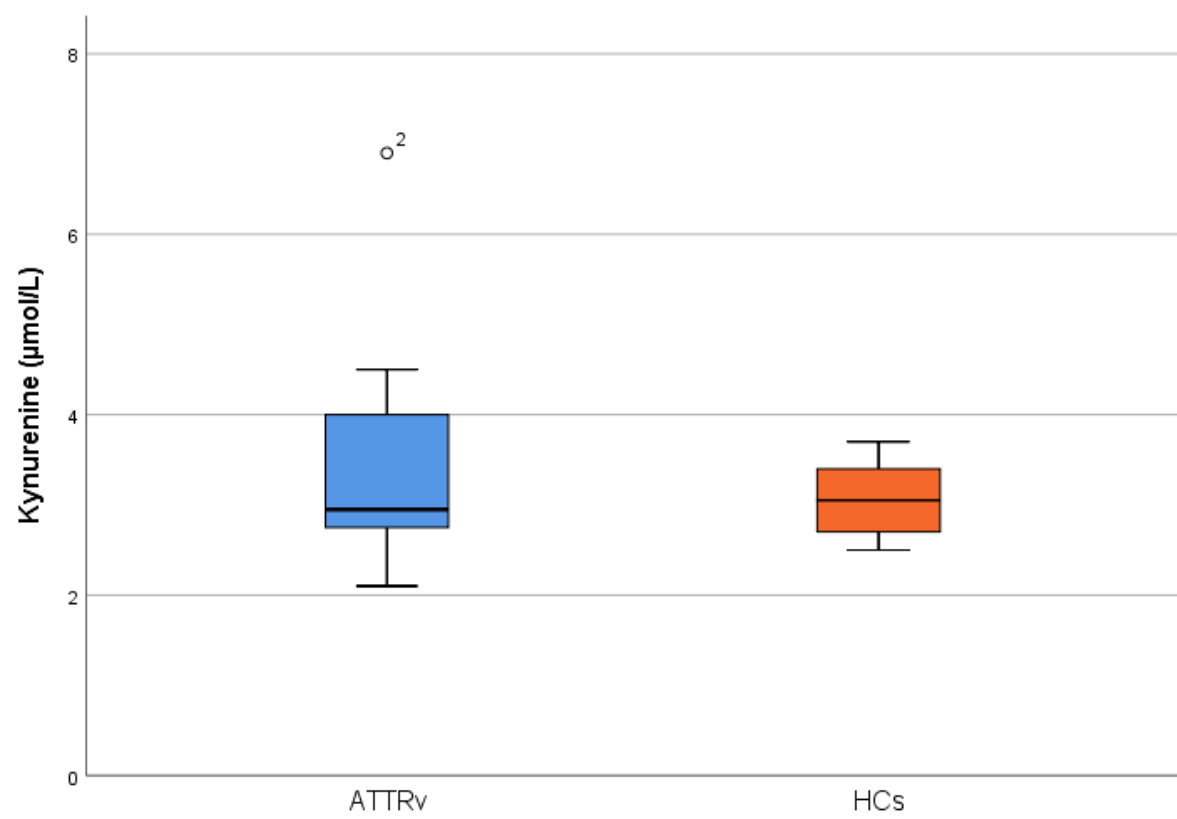

Supplement: Supplementary file 1 [file ijms-23-16133-s001.zip › Supplementary figure S2.pdf]
